# Nuclear PD-L1 compartmentalization suppresses tumorigenesis and overcomes immunocheckpoint therapy resistance in mice via histone macroH2A1

**DOI:** 10.1172/JCI181314

**Published:** 2024-11-15

**Authors:** Yong Liu, Zhi Yang, Shuanglian Wang, Rui Miao, Chiung-Wen Mary Chang, Jingyu Zhang, Xin Zhang, Mien-Chie Hung, Junwei Hou

**Affiliations:** 1 Department of Otolaryngology Head and Neck Surgery, Xiangya Hospital, Central South University, Changsha, China.; 2 Otolaryngology Major Disease Research Key Laboratory of Hunan Province, Changsha, China.; 3 Clinical Research Center for Pharyngolaryngeal Diseases and Voice Disorders in Hunan Province, Changsha, China.; 4 National Clinical Research Center for Geriatric Disorders,; 5 Xiangya Cancer Center, and; 6 Center for Molecular Oncology and Immunology, Xiangya Hospital, Central South University, Changsha, China.; 7 Institute of Biochemistry and Molecular Biology and; 8 Graduate Institute of Biomedical Sciences, Research Center for Cancer Biology and Center for Molecular Medicine, China Medical University, Taichung, Taiwan.

**Keywords:** Cell biology, Oncology, Cancer immunotherapy

## Abstract

Canonically PD-L1 functions as the inhibitory immune checkpoint on cell surface. Recent studies have observed PD-L1 expression in the nucleus of cancer cells. But the biological function of nuclear PD-L1 (nPD-L1) in tumor growth and antitumor immunity is unclear. Here we enforced nPD-L1 expression and established stable cells. nPD-L1 suppressed tumorigenesis and aggressiveness in vitro and in vivo. Compared with PD-L1 deletion, nPD-L1 expression repressed tumor growth and improved survival more markedly in immunocompetent mice. Phosphorylated AMPKα (p-AMPKα) facilitated nuclear PD-L1 compartmentalization and then cooperated with it to directly phosphorylate S146 of histone variant macroH2A1 (mH2A1) to epigenetically activate expression of genes of cellular senescence, JAK/STAT, and Hippo signaling pathways. Lipoic acid (LA) that induced nuclear PD-L1 translocation suppressed tumorigenesis and boosted antitumor immunity. Importantly, LA treatment synergized with PD-1 antibody and overcame immune checkpoint blockade (ICB) resistance, which likely resulted from nPD-L1–increased MHC-I expression and sensitivity of tumor cells to interferon-γ. These findings offer a conceptual advance for PD-L1 function and suggest LA as a promising therapeutic option for overcoming ICB resistance.

## Abstract 2

Nuclear compartmentalization of PD-L1 suppresses tumorigenesis and overcomes immune checkpoint therapy resistance via histone macroH2A1 phosphorylation

## Graphical abstract

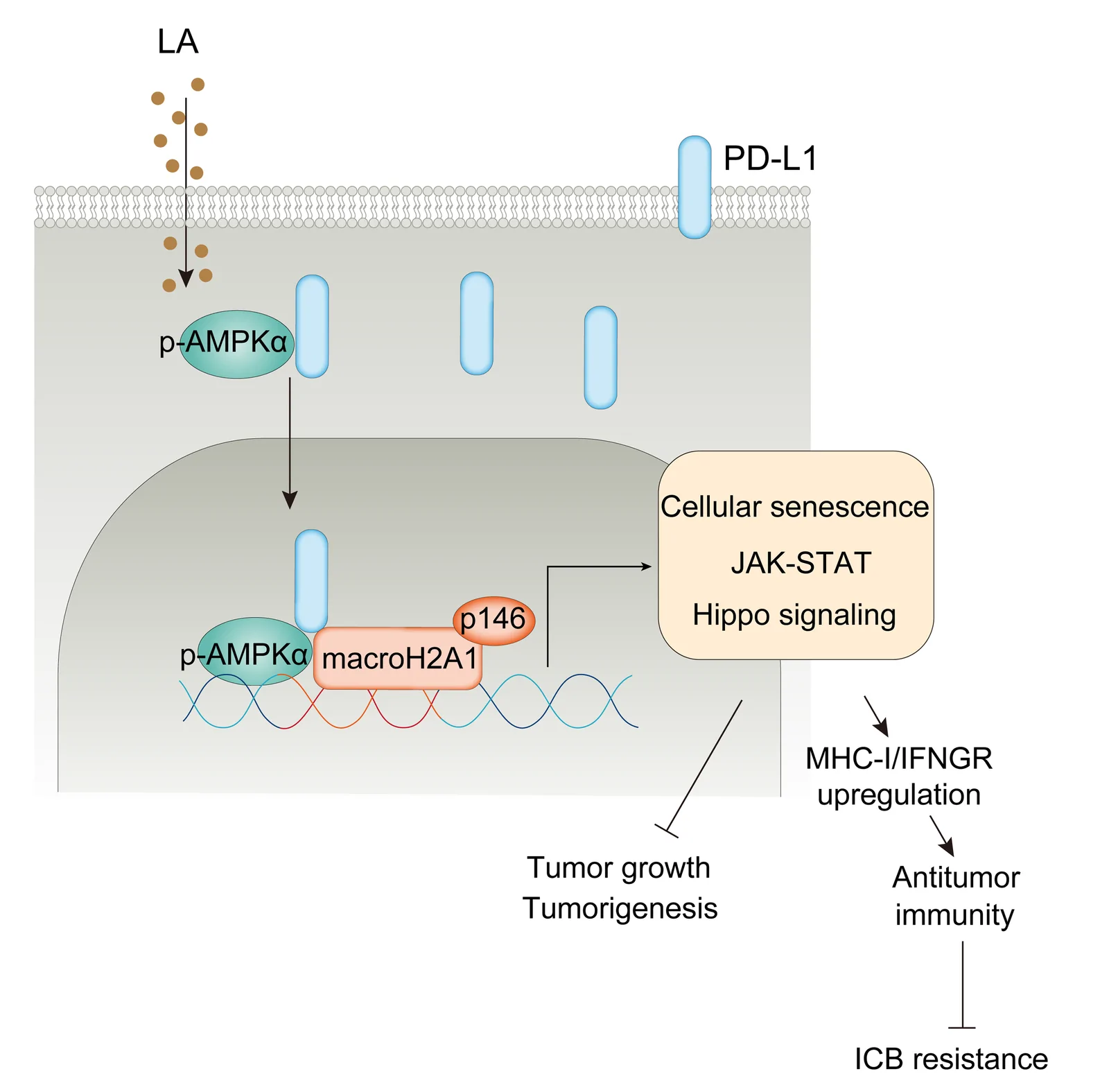

## Introduction

Cancer cells can evade attacks from cytotoxic lymphocytes through the interaction of programmed death ligand 1 (PD-L1) with its receptor, programmed cell death protein 1 (PD-1), on the cell surface (1). Antibodies targeting PD-1/PD-L1 have been used in a wide range of cancer types and substantially improve patient survival (2–5). However, the intrinsic and acquired resistance of PD-1/PD-L1 blockade therapy largely limits its efficacy in cancer patients (2, 6). Strategies to conquer PD-1/PD-L1 blockade therapy resistance are highly desirable and urgently needed in the clinic.

Although PD-L1 functions as an inhibitory immune checkpoint on the cell surface, its localization in the nucleus has been detected (7, 8). One study showed that the basal level of nuclear PD-L1 (nPD-L1) translocation depended on its acetylation, which limited the antitumor response to PD-1 blockade (7). However, normally the majority of PD-L1 locates in cytosol plasma and the basal level of nPD-L1 is extremely low (<3%) (8). Thus, the biological function of extensively accumulated nPD-L1 is still unclear. We previously screened the stress conditions, ligands, and 40 compounds for triggers of nuclear PD-L1 translocation and reported that hypoxia induces nuclear PD-L1 translocation and switches TNF-α–induced apoptosis to pyroptosis, which results in tumor necrosis (8). Owing to lack of a DNA-binding domain, PD-L1 interacts with transcriptional factors to activate gene expression (8). AMP-activated protein kinase (AMPK) activated by metformin or energy stress physically binds to PD-L1 to dictate its abundance on the cell membrane and promotes antitumor immunity (9, 10), suggesting that AMPK may serve as a potential target for immune checkpoint blockade (ICB) therapy.

The fundamental features of cancer include uncontrolled proliferation, apoptosis resistance, metastasis, and evasion of immune surveillance (11), all of which could be regulated through epigenetic mechanisms including histone modification (12). Histone variant macroH2A1 (mH2A1) has been reported as a tumor suppressor (12). mH2A1 expression suppressed cancer cell proliferation and anchorage-independent cell growth (13, 14). It also serves as a stem cell reprogramming barrier (15–17) and inhibits tumorigenicity (18). Moreover, mH2A1 contributes to cellular senescence, and enriched expression of mH2A1 was observed in liver cancer and premalignant lung adenomas that have a high percentage of senescent cells (19–22). Notably, a recent study revealed that loss of mH2A1 in liver cancer cells promoted regulatory T cell activation, suggestive of a role in antitumor immunity (23). Importantly, lack of mH2A1 predicted worse outcome and increased malignancy in cancer patients (13, 24). However, how mH2A1 epigenetically regulates tumor growth and antitumor immunity is unknown.

Lipoic acid (LA) as an antioxidant has become a common dietary supplement. In skeletal muscle, LA activates AMPK to increase insulin sensitivity (25, 26). In the last decade, LA has been shown to have an antitumor effect in multiple cancer types (27–30). However, the role of LA in antitumor immunity is still unclear.

## Results

### Nuclear compartmentalization of PD-L1 suppresses tumorigenesis and tumor growth in nude mice.

**Figure 1:**
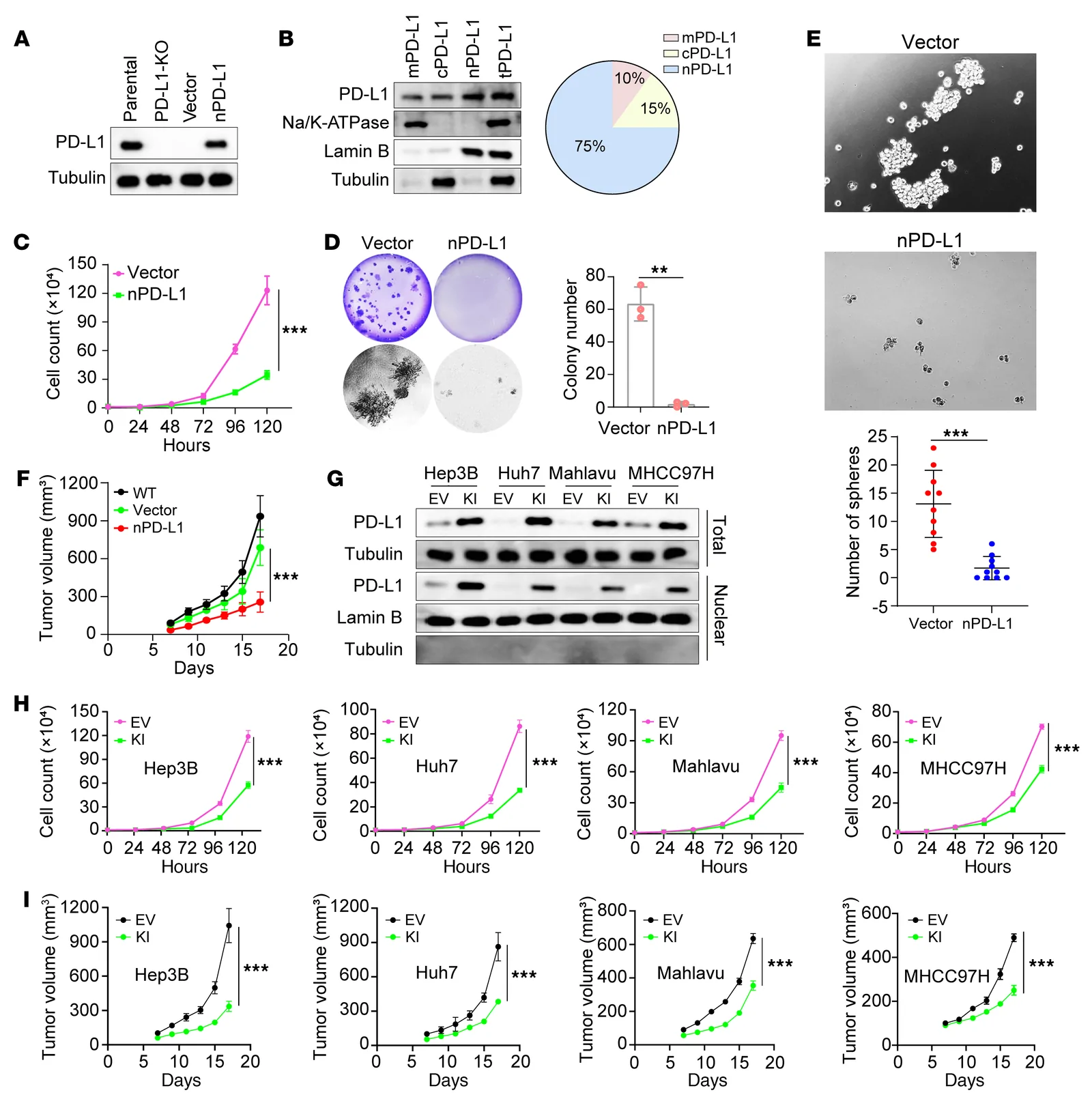
nPD-L1 suppressed tumorigenesis and aggressiveness. (**A**–**F**) Enforced expression of human nPD-L1 in Hep3B cells with endogenous PD-L1 knockout. (**A**) Detection of PD-L1 in parental and stable Hep3B cells. PD-L1-KO, PD-L1–knockout cells; Vector, PD-L1–knockout cells with empty vector expression; nPD-L1, PD-L1–knockout cells with re-expression of nPD-L1. (**B**) Analysis of the percentage of PD-L1 in membrane (mPD-L1), cytosol (cPD-L1), and nucleus (nPD-L1) by cellular fractionation. tPD-L1, total PD-L1. (**C**) Tumor cell growth (*n* = 3). (**D**) Anchorage-independent growth of tumor cells. Photomicrographs of representative colonies were taken. Colonies were then stained with crystal violet, and colonies larger than 100 μm were counted (*n* = 3). (**E**) Sphere-forming assay for tumor cells. Spheres larger than 50 μm were counted (*n* = 10). (**F**) Tumor growth in nude mice (*n* = 10 mice per group). (**G**–**I**) nPD-L1 knockin (KI) in Hep3B/Huh7/Mahlavu/MHCC97H cells. Empty vector (EV) was used as a negative control. (**G**) Immunoblotting of PD-L1 after cellular fractionation. (**H**) Tumor cell growth (*n* = 3). (**I**) Tumor growth in nude mice (*n* = 10 mice per group). Data shown are mean ± SD. Mann-Whitney test for **E** and Huh7 tumor growth in **I**, and unpaired 2-tailed *t* test for the rest. **P < 0.01, ***P < 0.001.

To investigate the physiological role of nPD-L1 in tumor growth, we enforced PD-L1 expression in the nucleus of Hep3B cells with endogenous PD-L1 loss (8) (Figure 1, A and B). nPD-L1 significantly slowed down tumor cell growth (Figure 1C) and inhibited colony-forming ability of tumor cells (Figure 1D). In addition, inhibition of aggressive growth pattern of tumor cells by nPD-L1 expression was also observed (Figure 1D). Similarly, single-cell-derived tumor sphere formation efficiency of Hep3B cells was dramatically inhibited (Figure 1E). A serial dilution and implantation of tumor cells in nude mice showed that nuclear PD-L1 compartmentalization caused remarkable reduction of tumor incidence (Table 1). Consistently, marked tumor shrinkage occurred in nPD-L1–overexpressed Hep3B tumors (Figure 1F). Next, we asked whether the antitumor effect of nPD-L1 is general. To answer this question, we established nPD-L1–knockin (KI) stable cells in Hep3B, Huh7, Mahlavu, and MHCC97H cell lines (Figure 1G). Expectedly, nPD-L1 KI suppressed tumor cell growth and caused tumor shrinkage in these cell lines (Figure 1, H and I). Together, these data suggested that nuclear compartmentalization of PD-L1 diminishes tumor initiation and growth.

### Nuclear compartmentalization of PD-L1 inhibits tumor growth and enhances antitumor immunity more markedly than endogenous PD-L1 loss in immunocompetent mice.

**Figure 2:**
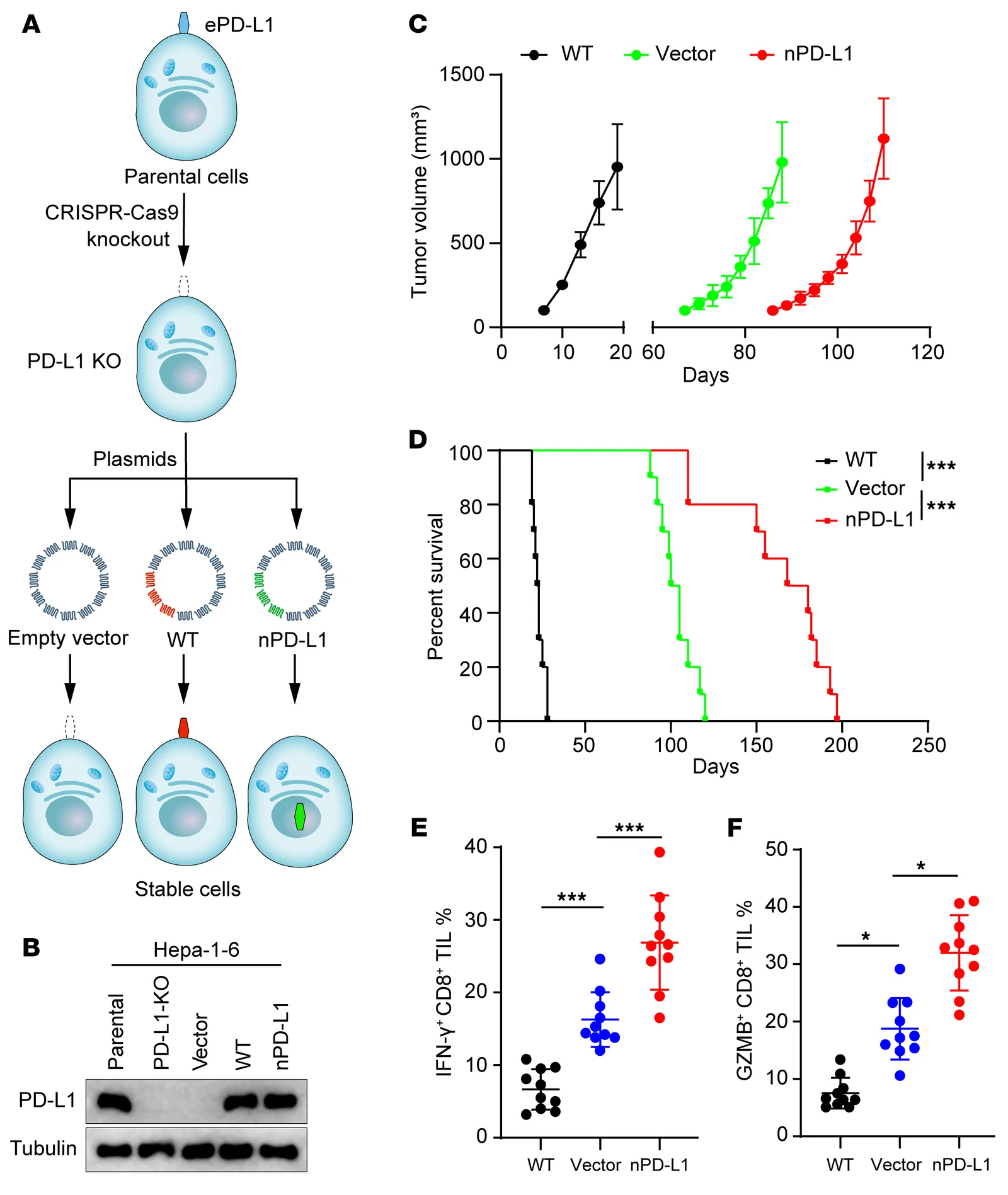
nPD-L1 elicited robust antitumor immunity in immunocompetent mice. (**A**) Schematic model for the strategy of establishment of Hepa-1-6 stable cells. Enforced expression of mouse nPD-L1 in Hepa-1-6 cells with endogenous PD-L1 (ePD-L1) deletion. Empty vector as a negative control for nPD-L1. Vector, Hepa-1-6-ePD-L1-KO-vector; WT, Hepa-1-6-ePD-L1-KO–wild-type PD-L1; nPD-L1, Hepa-1-6-ePD-L1-KO–nPD-L1. (**B**) Immunoblotting of PD-L1 in Hepa-1-6 stable cells in **A**. (**C**–**F**) Tumors of Hepa-1-6 stable cells in **A** in immunocompetent mice (*n* = 10 mice per group). (**C**) Tumor growth. (**D**) Overall survival (log-rank test). (**E**) Percentage of CD8^+^ tumor-infiltrating lymphocytes (TILs) expressing IFN-γ. (**F**) Percentage of CD8^+^ TILs expressing GZMB. TIL analysis was done via flow cytometry. Each dot represents a mouse in **E** and **F**. One-way ANOVA (Dunnett’s correction) for **E**; Kruskal-Wallis 1-way ANOVA (Dunn’s correction) for **F**. Data shown in **C**–**F** are mean ± SD and are representative of 3 independent experiments. *P < 0.05, ***P < 0.001.

To determine the role of nPD-L1 in antitumor immunity, we enforced nPD-L1 expression in Hepa-1-6 stable cells with endogenous PD-L1 loss as previously described (8) (Figure 2, A and B). As expected, deficiency of PD-L1 that inhibits cytotoxic T cell activity significantly delayed tumorigenesis and slowed down tumor growth, and consequently improved overall survival of mice compared with the group with wild-type PD-L1 expression (Figure 2, C and D). Surprisingly, more dramatic suppression of tumor growth and corresponding improvement of survival rates by nPD-L1 expression were observed in comparison with endogenous PD-L1 loss (Figure 2, C and D). This result evidenced that nuclear compartmentalization of PD-L1 had a stronger antitumor effect than PD-L1 loss and suggested that nPD-L1 expression may fuel antitumor immunity and overcome the resistance of tumor cells to PD-L1 inhibition. Supporting this, remarkably increased infiltration of cytotoxic CD8^+^ T cells with interferon-γ (IFN-γ) or granzyme B (GZMB) expression was recorded in Hepa-1-6 tumors with nPD-L1 expression (Figure 2, E and F, and Supplemental Figure 1; supplemental material available online with this article; https://doi.org/10.1172/JCI181314DS1). Thus, nuclear compartmentalization of PD-L1 may elicit potent antitumor immunity.

### LA induces nuclear PD-L1 translocation through p-AMPKα.

**Figure 3:**
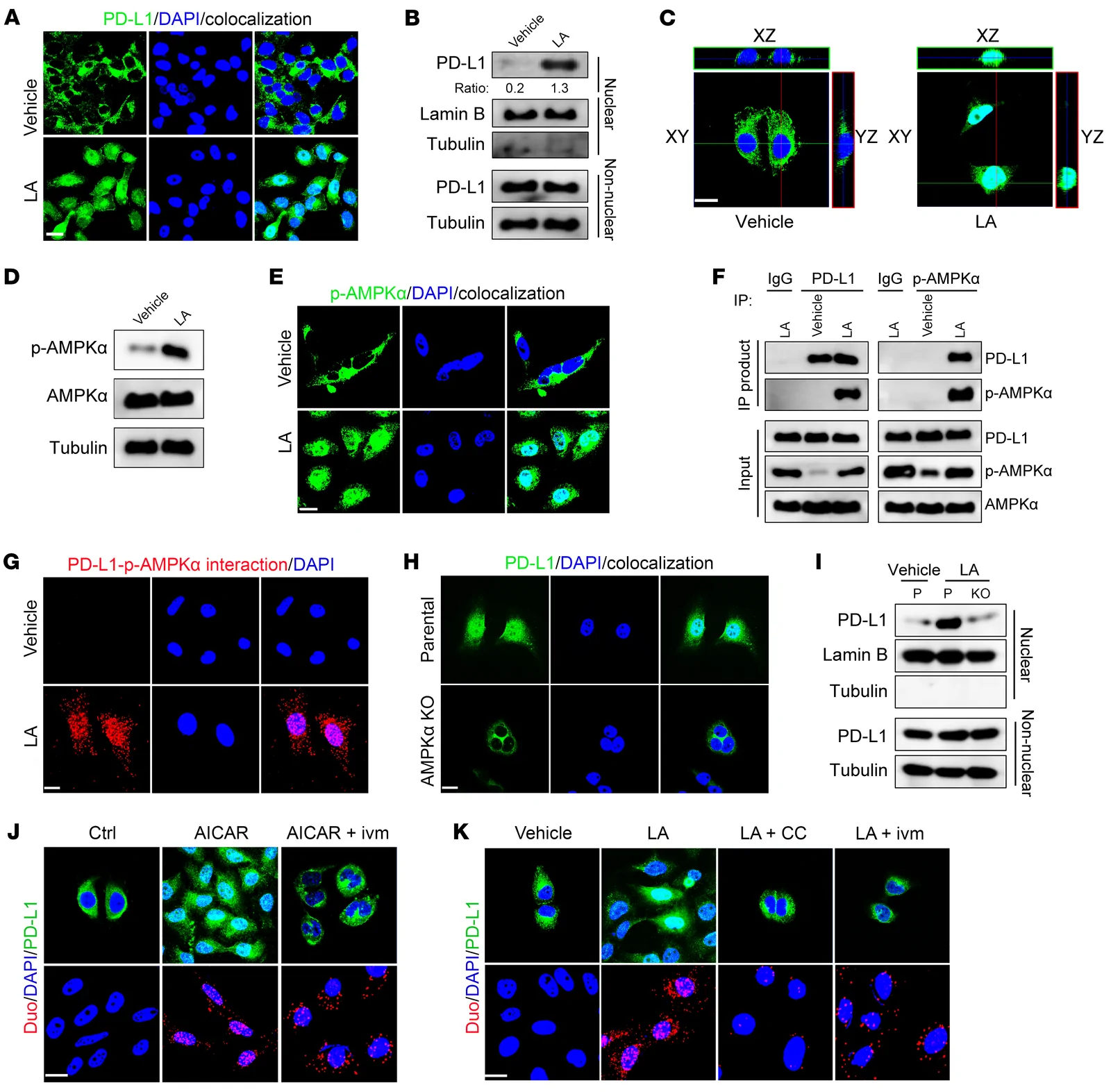
Lipoic acid induced nuclear compartmentalization of PD-L1 through AMPKα. (**A**) Confocal microscopy analysis of PD-L1 expression in MHCC97H cells treated with lipoic acid (LA) (200 μM). Scale bar: 20 μm. (**B**) Immunoblotting of PD-L1 after cellular fractionation in MHCC97H cells treated with LA (200 μM). (**C**) 3D visualization of nPD-L1 in MHCC97H cells treated with LA. Scale bar: 20 μm. (**D**) Immunoblotting of AMPKα and p-AMPKα in Hep3B cells treated with LA. (**E**) Confocal microscopy analysis of p-AMPKα expression in Hep3B cells treated with LA. Scale bar: 20 μm. (**F**) Immunoprecipitation (IP) and Western blot analysis of PD-L1/p-AMPKα interaction in Hep3B cells. (**G**) Duolink assay (red dots: interaction between p-AMPKα and PD-L1) with antibodies specific for p-AMPKα and PD-L1 in Hep3B cells treated with LA. Scale bar: 20 μm. (**H**) Confocal microscopy analysis of PD-L1 expression in LA-treated Hep3B cells with deletion of AMPKα. Scale bar: 20 μm. (**I**) Immunoblotting of PD-L1 after cellular fractionation in LA-treated Hep3B parental (P) or AMPKα-knockout (KO) cells. (**J**) Hep3B cells were treated with the AMPKα activator AICAR (500 μM) or the importin α/β inhibitor ivermectin (25 μM). Localization of PD-L1 or PD-L1/p-AMPKα interaction (red dots) was analyzed by confocal microscopy (top) and Duolink assay (bottom). Scale bar: 20 μm. (**K**) Hep3B cells were treated with LA, the p-AMPKα inhibitor compound C (CC), or ivermectin. Localization of PD-L1 or PD-L1/p-AMPKα interaction (red dots) was analyzed by confocal microscopy (top) and Duolink assay (bottom). Scale bar: 20 μm.

We screened for stimuli that may facilitate nuclear PD-L1 translocation (8). LA was shown by confocal analysis to induce strong nuclear translocation of PD-L1 in MHCC97H cells (Figure 3A), which was further validated by cellular fraction and 3D reconstruction of *Z*-stack images (Figure 3, B and C, and Supplemental Figure 2). No change was observed in PD-L1 expression level in MHCC97H and Hep3B cells with the treatment of LA for 48 hours (Supplemental Figure 3). To identify the key molecule that facilitates nuclear translocation of PD-L1, we performed coimmunoprecipitation (co-IP) of nPD-L1 and mass spectrum analysis of its interactome. In the nPD-L1–interacting protein list, we observed AMPKα (Supplemental Table 1). Studies reported physical interaction of phosphorylated AMPKα (p-AMPKα) with PD-L1 (9, 10). Next, we asked whether AMPKα favored nuclear translocation of PD-L1. We showed that LA treatment activated p-AMPKα and promoted its nuclear translocation (Figure 3, D and E). Co-IP analysis showed that p-AMPKα bound to PD-L1 in response to LA treatment (Figure 3F); and a large proportion of p-AMPKα/PD-L1 interaction was located in the nucleus of Hep3B cells treated with LA (Figure 3G). Nuclear localization of PD-L1 peaked after 48 hours under LA treatment (Supplemental Figure 4). We and other laboratories reported that AMPKα activated by metformin or energy stress promotes PD-L1 degradation and fuels antitumor immunity (9, 10). In this study, long-term treatment of LA reduced PD-L1 expression while short-term did not (Supplemental Figure 5). In a realistic tumor microenvironment PD-L1 degradation may be accompanied by nuclear PD-L1 translocation in response to LA treatment. Therefore, the antitumor effect of LA may come from multiple different mechanisms. We deleted AMPKα in Hep3B cells (Supplemental Figure 6A). AMPKα deletion stopped LA-induced nuclear PD-L1 translocation (Figure 3, H and I). Consistently, compound 911 and A-769662, two specific AMPKα activators, could also induce nPD-L1 expression, which was abolished by AMPKα deletion in Hep3B cells (Supplemental Figure 7), indicating that p-AMPKα favors nuclear PD-L1 translocation. We previously reported that importin α/β mediated nuclear PD-L1 translocation (8). Consistently, the importin α/β inhibitor ivermectin blocked nuclear PD-L1 translocation induced by the AMPKα activator AICAR or LA in Hep3B cells, and p-AMPKα/PD-L1 interaction was trapped outside the nucleus (Figure 3, J and K). We further validated the LA-induced p-AMPKα/PD-L1 interaction and nuclear PD-L1 translocation in Mahlavu cells, and similar results were observed (Supplemental Figure 8). Taken together, these data suggested that nuclear translocation of PD-L1 requires LA-mediated p-AMPKα activation.

### LA suppresses tumorigenesis via nuclear PD-L1 compartmentalization.

**Figure 4:**
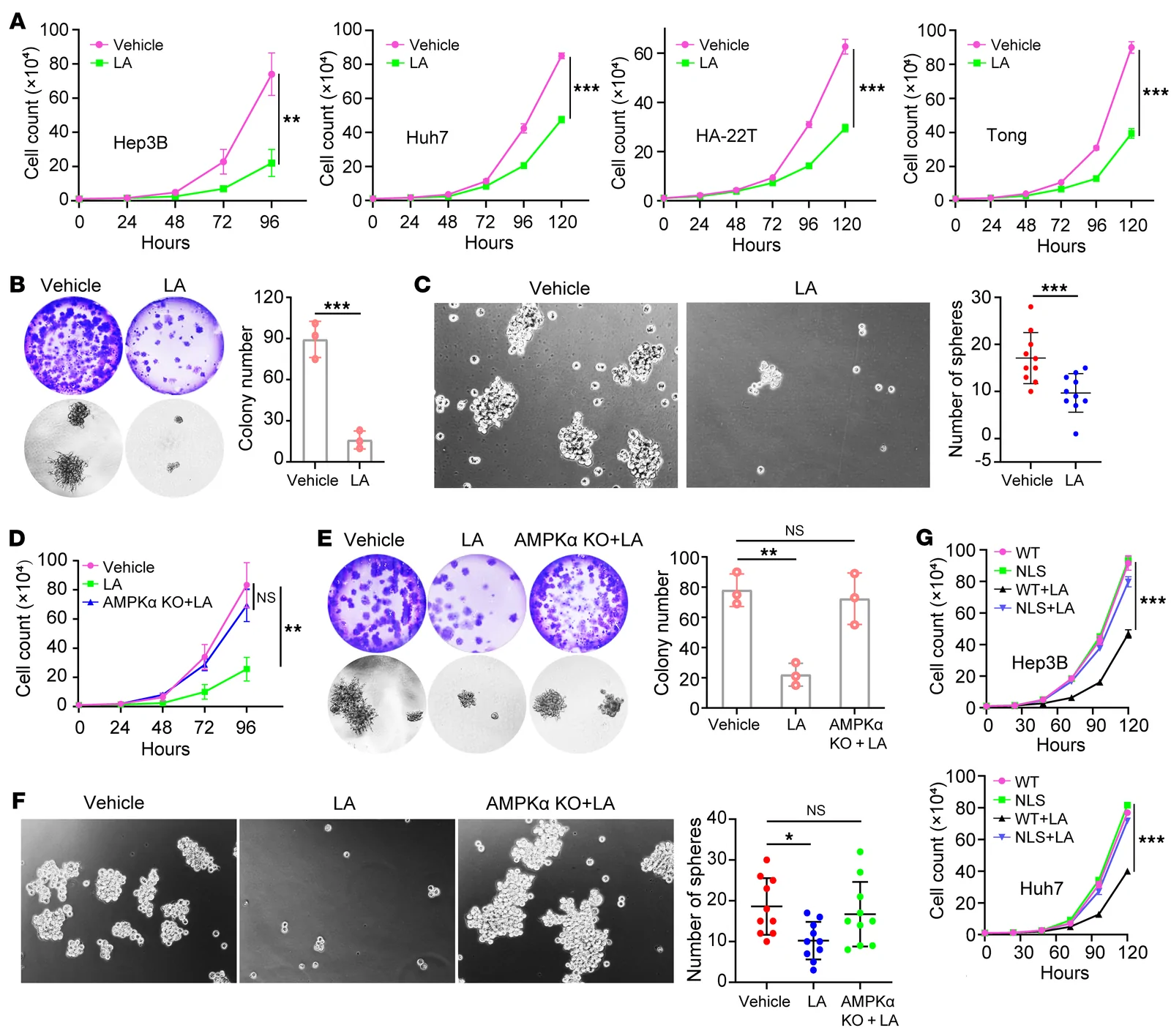
LA suppressed tumorigenesis and aggressiveness through nuclear compartmentalization of PD-L1. (**A**) Growth analysis of Hep3B/Huh7/HA-22T/Tong cells treated with LA (200 μM) (*n* = 3). (**B** and **C**) Hep3B cells were treated with LA (200 μM). (**B**) Anchorage-independent growth of tumor cells (*n* = 3). Colonies were taken, stained, and counted as in Figure 1B. (**C**) Sphere-forming assay for tumor cells (*n* = 10). Spheres were taken and counted as in Figure 1C. (**D**–**F**) Hep3B parental or AMPKα-knockout (AMPKα KO) cells were treated with LA (200 μM). Tumor cell growth (*n* = 3) (**D**), anchorage-independent growth (*n* = 3) (**E**), and tumor sphere formation (*n* = 10) (**F**) were analyzed as in Figure 1, A–D. (**G**) Enforced expression of human wild-type (WT) and nuclear localization signal–mutated (NLS-mutated) PD-L1 in Hep3B and Huh7 cells with endogenous PD-L1 loss. Cells were treated with LA (200 μM) and subjected to growth analysis (*n* = 3). Data shown are mean ± SD. Unpaired 2-tailed *t* test for **A**–**C** and **G**; 1-way ANOVA (Dunnett’s correction) for **D**–**F**. *P < 0.05, **P < 0.01, ***P < 0.001.

LA has been shown to inhibit the proliferation of cancer cells (27, 29, 30). We showed that LA significantly suppressed tumor cell growth in Hep3B, Huh7, HA-22T, and Tong cells (Figure 4A), and inhibited colony-forming ability as well as aggressiveness (Figure 4B) of Hep3B cells. Furthermore, remarkable reduction of tumor spheres occurred in Hep3B cells treated with LA (Figure 4C). To study the role of nPD-L1 in LA-induced tumor suppression, we tested the antitumor effect of LA in Hep3B cells with AMPKα deletion that could block nuclear PD-L1 translocation. AMPKα loss dramatically ablated LA-induced suppression of tumor cell growth (Figure 4D), colony-forming ability as well as aggressiveness (Figure 4E), and tumor sphere formation (Figure 4F). As expected, tumor incidence reduction induced by LA treatment in nude mice was abolished by AMPKα loss (Table 2). In addition, we mutated the nuclear localization sequence (NLS) of PD-L1 to prevent its translocation into the nucleus (8) and stably overexpressed wild-type and NLS-mutated PD-L1 in Hep3B and Huh7 cells with endogenous PD-L1 deletion. We found that NLS mutation of PD-L1 significantly ablated LA-induced suppression of tumor cell growth (Figure 4G), providing direct evidence that nPD-L1 contributes to LA-induced tumor suppression. These data suggest that LA-induced suppression of tumorigenesis requires p-AMPKα–mediated nuclear PD-L1 compartmentalization.

### LA-mediated nuclear PD-L1 compartmentalization contributes to tumor growth repression in a T cell–dependent and –independent manner.

**Figure 5:**
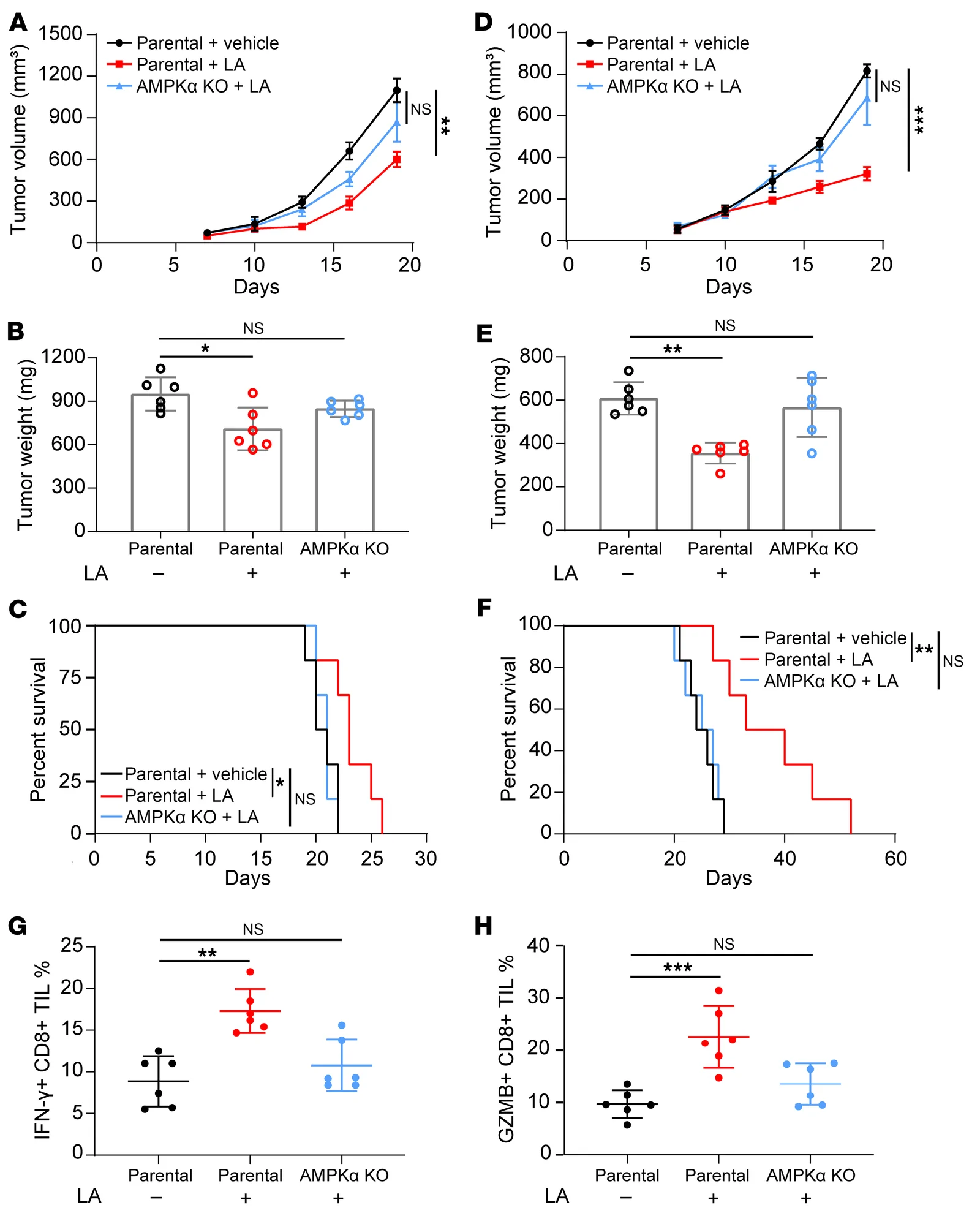
LA enhanced antitumor immunity through nuclear compartmentalization of PD-L1. (**A**–**C**) Nude mice bearing Hepa-1-6 or Hepa-1-6-AMPKα KO tumors were orally treated with LA (50 mg/kg/d) (*n* = 6 mice per group). Tumor growth (**A**), tumor weight (**B**), and mouse survival (log-rank test) (**C**) were analyzed. (**D**–**H**) C57BL/6 immunocompetent mice bearing Hepa-1-6 or Hepa-1-6-AMPKα KO tumors were orally treated with LA (50 mg/kg/d) (*n* = 6 mice per group). Tumor growth (**D**), tumor weight (**E**), mouse survival (log-rank test) (**F**), and percentage of CD8^+^ TILs expressing IFN-γ (**G**) or GZMB (**H**) in tumors were analyzed. TIL analysis was done via flow cytometry. Each dot represents a mouse in **G** and **H**. Data shown are mean ± SD and are representative of 3 independent experiments. One-way ANOVA (Dunnett’s correction) for **A**, **B**, **D**, and **H**; Kruskal-Wallis 1-way ANOVA (Dunn’s correction) for **E** and **G**. *P < 0.05, **P < 0.01, ***P < 0.001.

To investigate the impact of LA-induced nuclear PD-L1 compartmentalization on antitumor immunity, we knocked out AMPKα to prevent nuclear translocation of PD-L1 in mouse Hepa-1-6 cells (Supplemental Figure 6B). Although LA treatment repressed tumor growth (Figure 5A), reduced tumor weight (Figure 5B), and improved survival (Figure 5C) in nude mice bearing Hepa-1-6 tumors, AMPKα loss ablated this antitumor effect of LA, indicating that the antitumor function of LA requires nuclear PD-L1 compartmentalization in the absence of T cells. Notably, ablation of the antitumor effect of LA by AMPKα loss was augmented in immunocompetent mice compared with that in nude mice, which lack mature T cells (Figure 5, D–F), suggesting a role of T lymphocytes. Supporting this, the proportion of CD8^+^ tumor-infiltrating lymphocytes (TILs) expressing IFN-γ or GZMB, which was increased by LA treatment, was markedly decreased by AMPKα loss in immunocompetent mice (Figure 5, G and H, and Supplemental Figure 9). Therefore, we speculate that T cells may play a more important role in LA-mediated antitumor effect via nuclear PD-L1 compartmentalization.

### nPD-L1 may activate expression of genes of cellular senescence, JAK/STAT, and Hippo signaling pathways.

**Figure 6:**
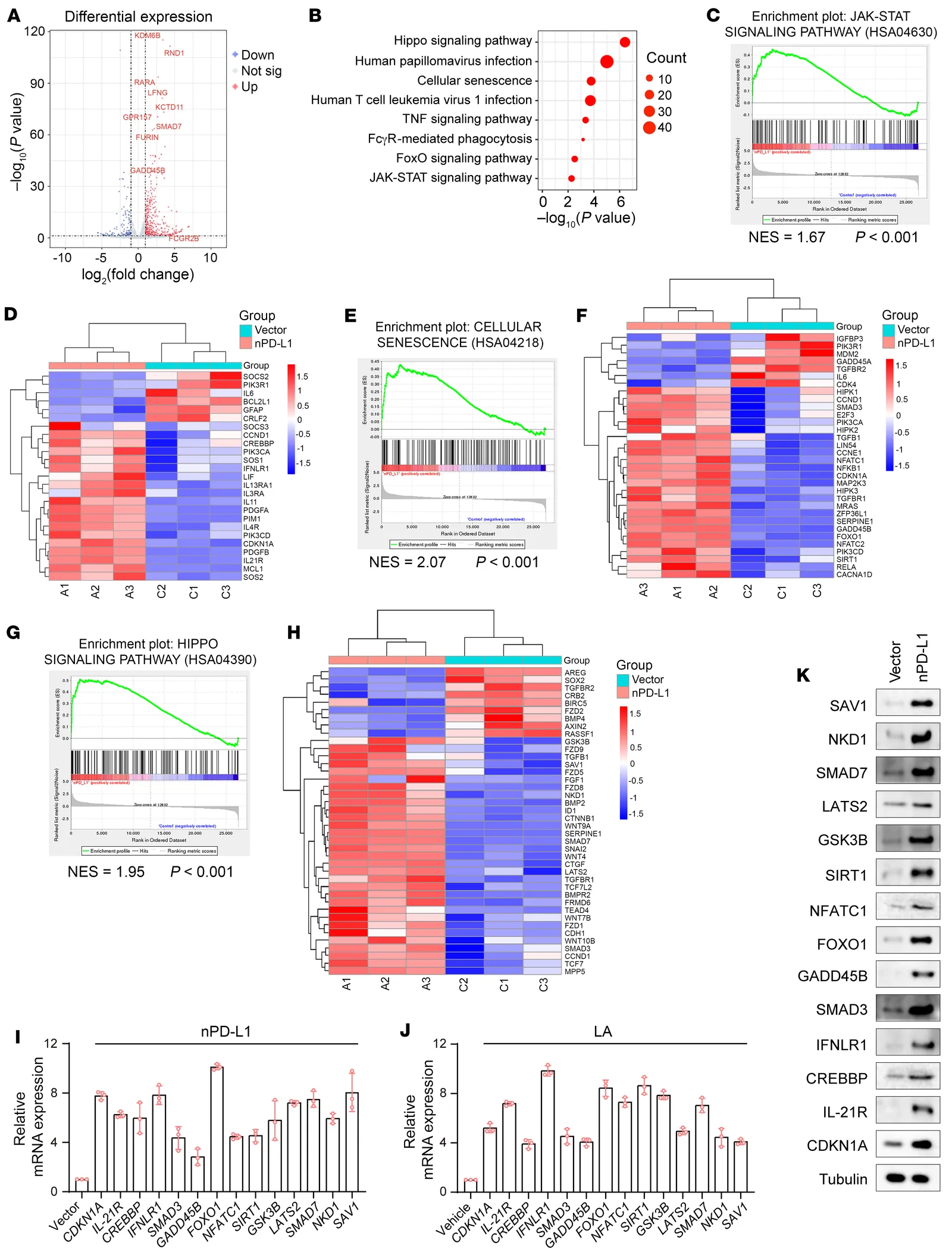
nPD-L1 may activate expression of genes of cellular senescence, JAK/STAT, and Hippo signaling pathways. (**A**) Differential expression of genes (nPD-L1 vs. vector) in Hep3B cells. Up, upregulated; Down, downregulated; Not sig, not significant. Genes that promote antitumor immunity are indicated. (**B**) Top 8 enriched KEGG pathways of upregulated genes upon nuclear PD-L1 compartmentalization in Hep3B cells (*n* = 3 independent sequenced samples per group). Modified Fisher’s exact test with Benjamini-Hochberg correction. Dot size indicates fold of enrichment. (**C**) GSEA signature analysis of enriched gene expression of JAK/STAT pathway by nPD-L1 in Hep3B cells (*n* = 3 independent sequenced samples per group). Kolmogorov-Smirnov test. (**D**) Heatmap display of downregulated or upregulated genes of JAK/STAT pathway by nPD-L1 in Hep3B cells. (**E**) GSEA signature analysis of enriched gene expression of cellular senescence pathway by nPD-L1 in Hep3B cells (*n* = 3 independent sequenced samples per group). Kolmogorov-Smirnov test. (**F**) Heatmap display of downregulated or upregulated genes of cellular senescence pathway by nPD-L1 in Hep3B cells. (**G**) GSEA signature analysis of enriched gene expression of Hippo signaling pathway by nPD-L1 in Hep3B cells (*n* = 3 independent sequenced samples per group). Kolmogorov-Smirnov test. (**H**) Heatmap display of downregulated or upregulated genes of Hippo signaling pathway by nPD-L1 in Hep3B cells. (**I** and **J**) Quantitative reverse transcription PCR (RT-PCR) analysis of the indicated genes in Hep3B cells expressing vector and nPD-L1 (**I**) or treated with LA (200 μM) (**J**). (**K**) Protein level of genes in **I**. Data are shown as the mean ± SD of *n* = 3 independent experiments.

To further explore the mechanisms by which nPD-L1 inhibits tumor growth and enhances antitumor immunity, we performed RNA sequencing analysis to compare gene expression profiles between nPD-L1–overexpressed and vector groups in Hep3B cells with endogenous PD-L1 deletion. nPD-L1 reduced the expression of 646 genes, and increased the expression of 1,398 genes, of which multiple ones have been shown to promote antitumor immunity (Figure 6A). Kyoto Encyclopedia of Genes and Genomes (KEGG) pathway enrichment analysis showed that nPD-L1–upregulated genes were involved in Hippo signaling, cellular senescence, and JAK/STAT signaling pathways (Figure 6B), which was further confirmed by gene set enrichment analysis (GSEA) signatures and heatmap analysis (Figure 6, C–H). Supporting this, we found that both mRNA and protein expression level of the critical genes of the 3 pathways was indeed increased by nPD-L1 and LA treatment (Figure 6, I–K). Thus, Hippo signaling, cellular senescence, and JAK/STAT pathways might be involved in the antitumor effect of nPD-L1.

### nPD-L1 cooperates with p-AMPKα to phosphorylate S146 of mH2A1 to epigenetically activate gene expression.

**Figure 7:**
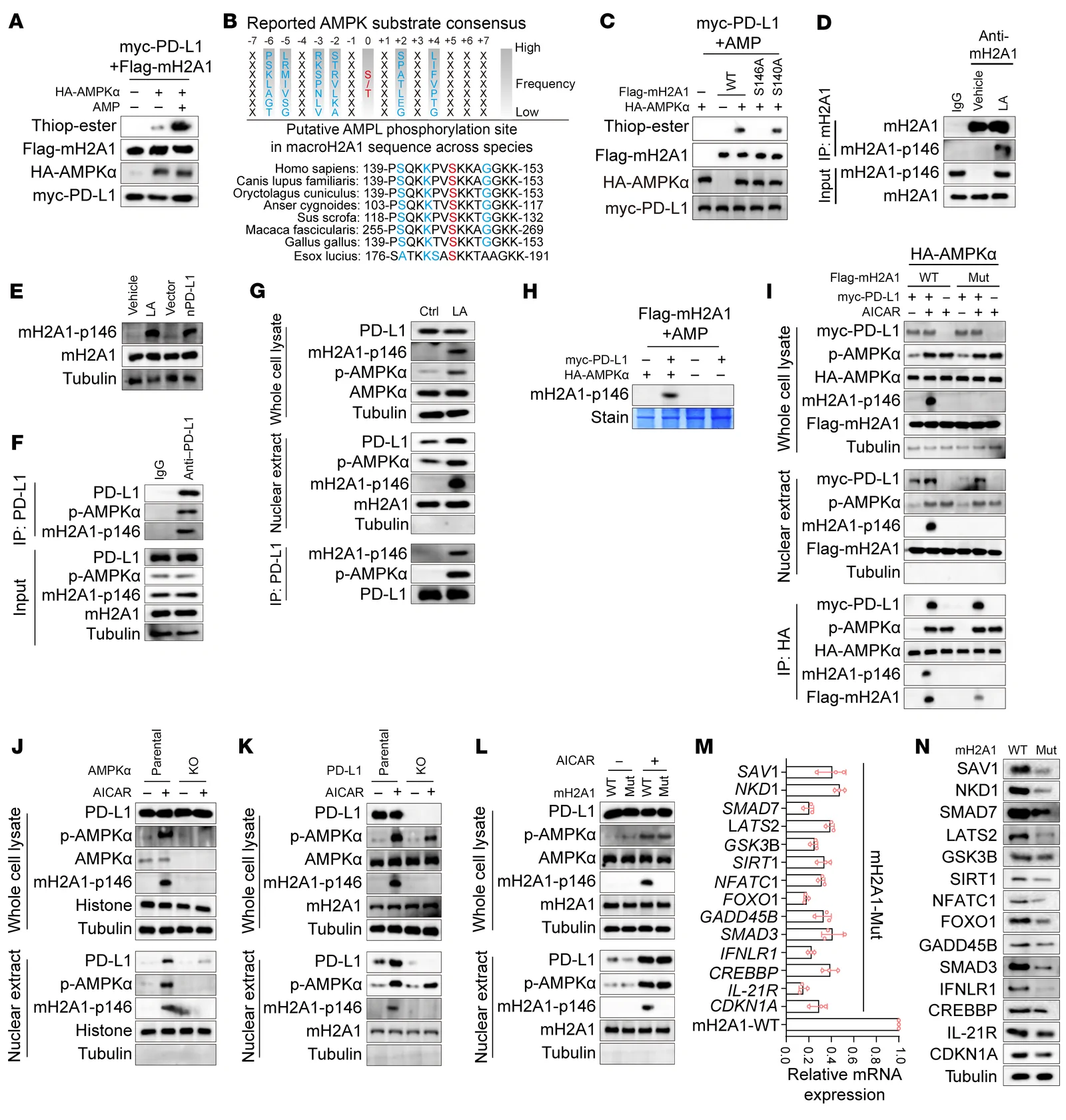
nPD-L1 cooperated with p-AMPKα to phosphorylate S146 of mH2A1 to epigenetically activate gene expression. (**A**) In vitro phosphorylation of histone mH2A1 by AMPKα was performed. Anti–thiophosphate ester antibody was used to detect mH2A1 phosphorylation. (**B**) AMPK phospho-motif of mH2A1 and conservative amino acid sequence across species. (**C**) In vitro phosphorylation of wild-type (WT) and mutant mH2A1. S140A was used as a negative control. (**D**) Co-IP analysis of mH2A1 phosphorylation at the site S146 in Hep3B cells treated with LA (1 mM). (**E**) Immunoblotting of mH2A1-p146 in LA-treated Hep3B cells and Hep3B-PD-L1-KO-nPD-L1 stable cells. (**F**) Co-IP analysis of the interactions of mH2A1-p146, PD-L1, and p-AMPKα. (**G**) Analysis of LA-induced activation of AMPKα, mH2A1, and nPD-L1 and their interaction by co-IP and cellular fraction in Hep3B cells. (**H**) In vitro phosphorylation of mH2A1 at the site S146 by the cooperation of PD-L1 and p-AMPKα. (**I**) Cellular fraction and co-IP analysis of mH2A1-p146 phosphorylation in 293T cells expressing genes as indicated. AICAR treatment (500 μM, 24 hours). (**J**) Cellular fraction analysis of PD-L1/p-AMPKα–induced mH2A1-p146 phosphorylation in Hep3B-PD-L1-KO stable cells treated with AICAR. (**K**) Cellular fraction analysis of PD-L1/p-AMPKα–induced mH2A1-p146 phosphorylation in Hep3B-AMPKα-KO stable cells treated with AICAR. (**L**) Cellular fraction analysis of AICAR-induced mH2A1-p146 phosphorylation in Hep3B cells with overexpression of wild-type (WT) or S146A-mutated (Mut) mH2A1. (**M**) Quantitative RT-PCR analysis of the indicated genes from mH2A1-WT or mH2A1-Mut groups in Hep3B cells. (**N**) Protein level of genes in **M**. Data shown are mean ± SD. Thiop-ester, thiophosphate ester.

Alteration of transcriptional levels of massive genes is most suggestive of epigenetic changes. AMPKα was reported to promote stress-induced gene transcription via histone phosphorylation (31). In nPD-L1 interactome, we observed histone variant mH2A1 (Supplemental Table 1). Thus, we investigated whether AMPKα could directly phosphorylate mH2A1. In vitro kinase assay showed that mH2A1 could be evidently phosphorylated by AMPKα in the presence of PD-L1 (Figure 7A). Based on the prediction database of phospho-motif of AMPKα (https://github.com/BrunetLabAMPK/AMPK_motif_analyzer) (32), a putative AMPKα phosphorylation site S146 in mH2A1, was identified (Figure 7B), which was also observed by mass spectrometric analysis (Supplemental Table 2). Consistently, S146A mutation of mH2A1 largely diminished its phosphorylation by AMPKα in vitro (Figure 7C). Hence, we generated and validated a specific antibody against mH2A1-p146 (Supplemental Figure 10). Immunoprecipitation analysis with the antibody revealed that LA indeed induced mH2A1-p146 (Figure 7D). Both LA treatment and nPD-L1 compartmentalization increased mH2A1-p146 level in Hep3B cells (Figure 7E). Furthermore, PD-L1 physically interacted with p-AMPKα and mH2A1-p146 in Hep3B cells (Figure 7F). LA treatment increased the expression level of p-AMPKα and mH2A1-p146 in both total cell lysate and nuclear extract and the interaction of PD-L1, p-AMPKα, and mH2A1-p146 (Figure 7G). In vitro kinase assay showed that p-AMPKα indeed could phosphorylate mH2A1 at its S146 site, which required PD-L1 assistance (Figure 7H). Supporting this, transfected HA-AMPKα, myc–PD-L1, and FLAG-mH2A1 associated in 293T cells in response to the AMPK agonist AICAR, and mH2A1-p146 was observed in the immunoprecipitation product (Figure 7I). The interaction and mH2A1-p146 phosphorylation were ablated by the mH2A1 S146A mutation and PD-L1 deficiency (Figure 7I), suggesting that PD-L1 is critical for the interaction of p-AMPKα and mH2A1 and mH2A1-p146 phosphorylation. Moreover, deletion of AMPKα abolished the nuclear translocation of PD-L1 and mH2A1-p146 phosphorylation in Hep3B cells treated with AICAR (Figure 7J), and lack of PD-L1 suppressed mH2A1-p146 phosphorylation independent of p-AMPKα nuclear translocation (Figure 7K), indicating the critical role of PD-L1 and p-AMPKα in mH2A1-p146 phosphorylation. Expectedly, the mH2A1 S146A mutation ablated AICAR-induced mH2A1-p146 phosphorylation (Figure 7L) and suppressed the transcription and protein expression of genes (Figure 7, M and N) in Hep3B cells. Taken together, these data suggested that nPD-L1 worked together with p-AMPKα to fuel mH2A1-p146 phosphorylation to epigenetically activate gene expression.

### LA treatment overcomes de novo and acquired resistance of tumor cells to ICB therapy.

**Figure 8:**
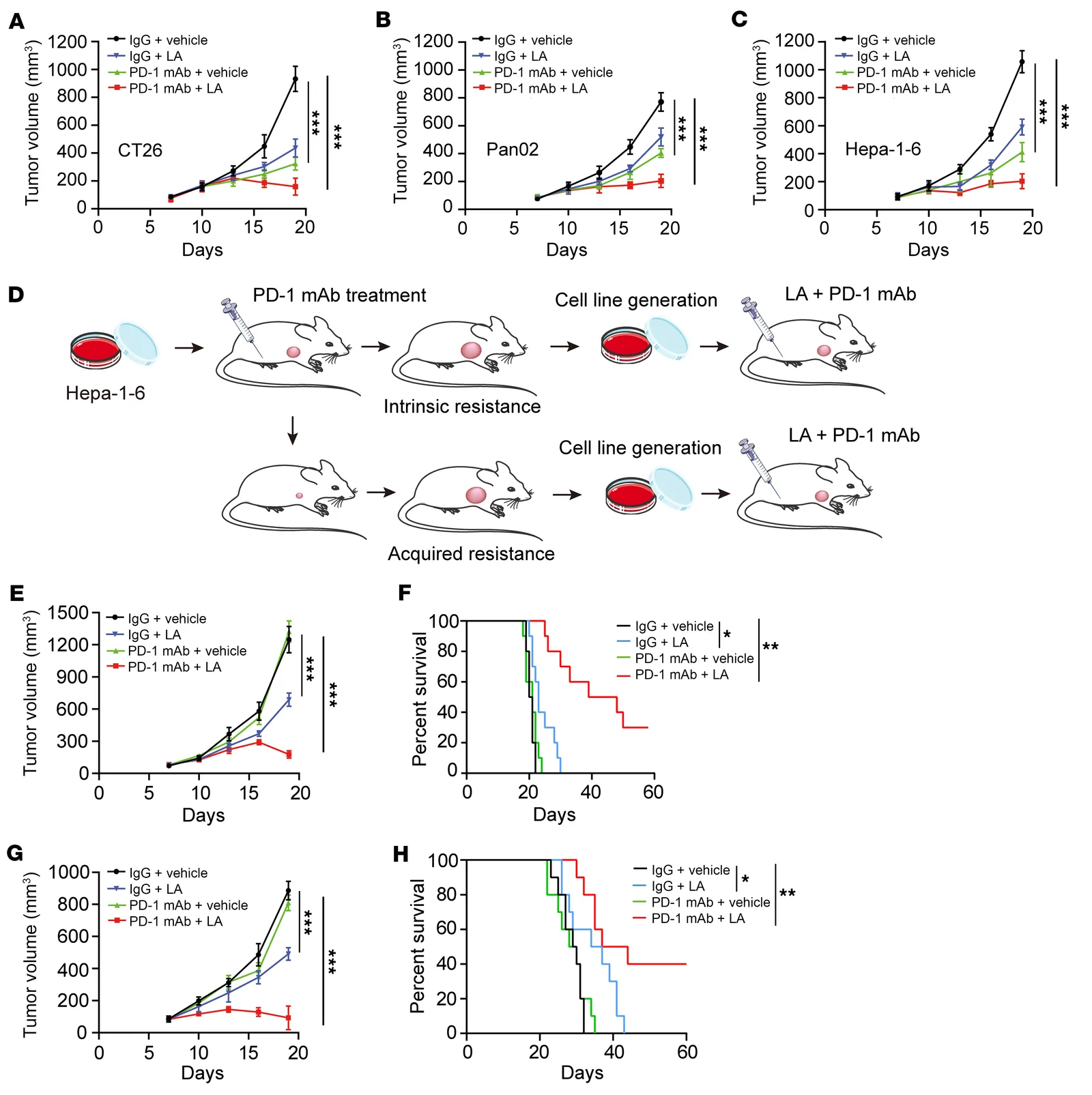
LA overcame the intrinsic and acquired resistance to ICB. (**A**–**C**) Immunocompetent mice bearing CT26 (**A**), Pan02 (**B**), or Hepa-1-6 (**C**) were orally treated with LA (50 mg/kg/d) or PD-1 antibody alone or in combination (*n* = 6 mice per group). (**D**) Schematic model of generation and combination treatment of Hepa-1-6 tumors with intrinsic and acquired resistance to PD-1 antibody in C57BL/6 mice. (**E**) Immunocompetent C57BL/6 mice bearing Hepa-1-6 tumors with intrinsic resistance to PD-1 antibody as shown in **D** were treated with LA or PD-1 antibody alone or in combination (*n* = 10 mice per group). (**F**) Survival analysis of mice in **E** (log-rank test). (**G**) Immunocompetent C57BL/6 mice bearing Hepa-1-6 tumors with acquired resistance to PD-1 antibody as shown in **D** were treated with LA or PD-1 antibody alone or in combination (*n* = 10 mice per group). (**H**) Survival analysis of mice in **G** (log-rank test). Data shown are mean ± SD. One-way ANOVA (Dunnett’s correction) for **A**–**C** and **G**; Brown-Forsythe 1-way ANOVA (Dunnett’s T3 correction) for **E**. *P < 0.05, **P < 0.01, ***P < 0.001.

Although PD-L1 deletion dramatically delayed tumorigenesis, rapid growth of Hepa-1-6 tumors with PD-L1 deficiency after tumors developed was observed (Figure 2C), indicating the resistance of tumor cells to PD-L1 target engagement. However, nuclear PD-L1 compartmentalization elicited stronger antitumor immunity and antitumor effect than PD-L1 deletion (Figure 2), indicating that nPD-L1 may overcome resistance of tumor cells to PD-L1 loss. Therefore we hypothesized that induction of nuclear PD-L1 translocation by LA may sensitize tumor cells to ICB therapy. Indeed, LA synergized with PD-1 mAb to inhibit growth of CT26, Pan02, and Hepa-1-6 tumors (Figure 8, A–C). Hepa-1-6 tumors that did not respond to PD-1 mAb treatment were collected, cultured, and then implanted into immunocompetent mice to generate the intrinsic resistance model of ICB, and the ones that initially responded but eventually acquired resistance to PD-1 mAb treatment were used to generate the acquired resistance model of ICB (Figure 8D). Notably, LA slowed down the growth of PD-1 mAb–resistant tumors and sensitized resistant tumor cells to PD-1 mAb (Figure 8E), and improved mouse survival (Figure 8F). Massive tumor shrinkage occurred (Figure 8G) and mouse survival was remarkably prolonged (Figure 8H) after LA combined with PD-1 mAb treatment. We further validated the role of p-AMPKα and nPD-L1 in ICB therapy in parental and ICB-resistant Hepa-1-6 tumors using A-769662 instead of LA, and similar results were observed (Supplemental Figure 11). Taken together, these data suggested that LA overcomes intrinsic and extrinsic resistance of tumor cells to PD-1 mAb treatment.

### nPD-L1 increases MHC-I expression and sensitizes tumor cells to IFN-γ.

**Figure 9:**
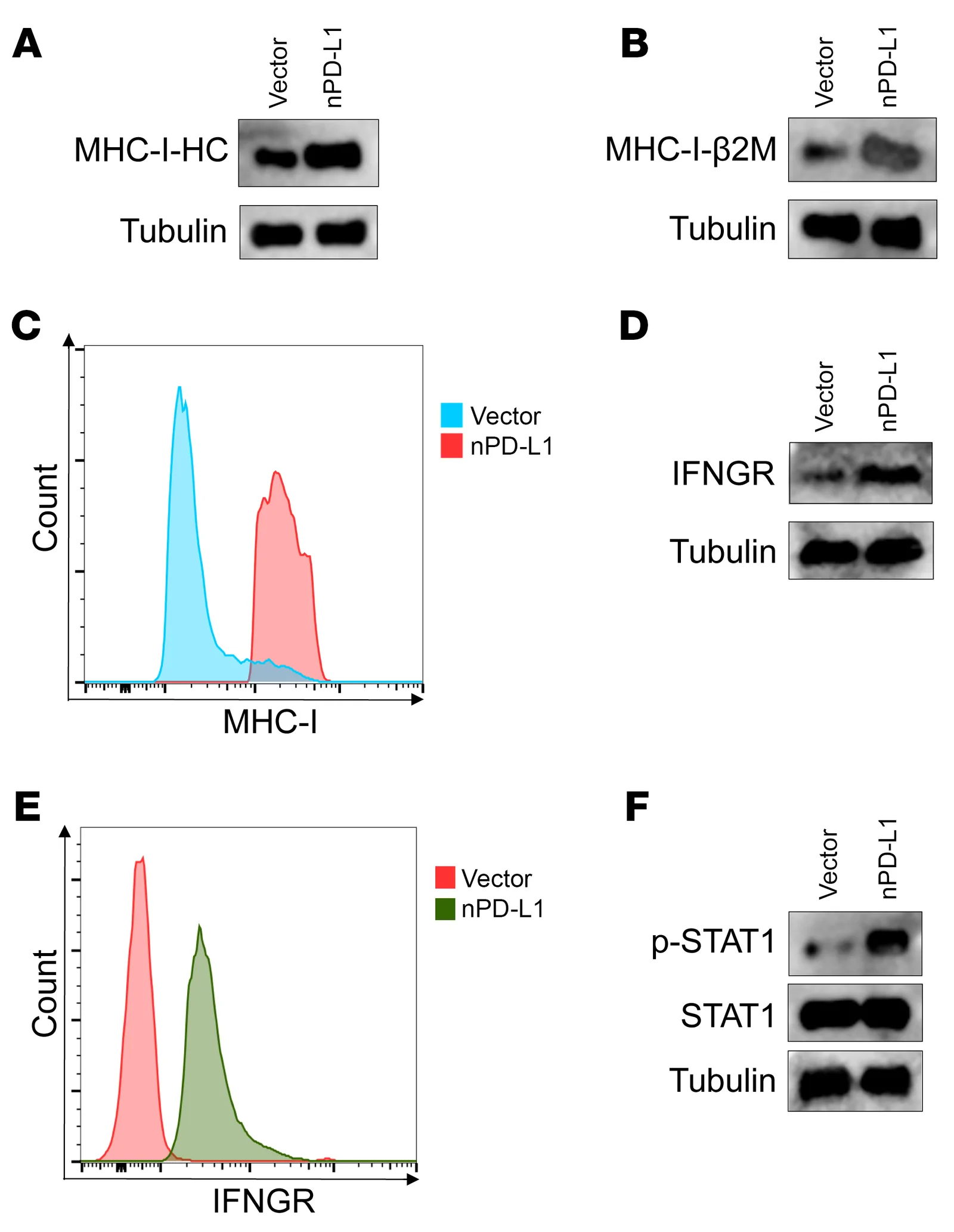
nPD-L1 increased the expression level of MHC-I and IFNGR in tumor cells and sensitized tumor cells to IFN-γ. (**A** and **B**) Immunoblotting of MHC-I heavy chain (MHC-I-HC) (**A**) and MHC-I light chain (MHC-I-β2M) (**B**) in Hep3B stable cells as indicated in Figure 1C. (**C**) The expression level of MHC-I protein on cell surface. (**D**) Immunoblotting of IFNGR. (**E**) The expression level of IFNGR protein on cell surface. (**F**) Immunoblotting of p-STAT1 in Hep3B stable cells treated with IFN-γ (10 ng/mL) for 2 hours.

nPD-L1 activated JAK/STAT, cellular senescence, and Hippo signaling pathways in tumor cells (Figure 6). The IFN-γ/JAK/STAT pathway plays a pivotal role in ICB therapy and antitumor immunity, and mutations or loss of the pathway genes caused resistance to ICB in cancer patients (33–37). Hippo signaling controlled the expression of genes of the MHC-I antigen processing and presentation pathway, and loss of LATS1 and LATS2, critical kinases required for Hippo signaling activation, favors tumor immune evasion through decreasing MHC-I expression (38, 39). In particular, cellular senescence upregulated the IFN-γ receptor (IFNGR) and enhanced MHC-I machinery via remodeling of the cell surface proteome in tumor cells, which hypersensitized tumor cells to IFN-γ and induced robust antigen presentation (40, 41). Therefore, we hypothesized that nPD-L1 may enhance the MHC-I expression and cellular response to IFN-γ in tumor cells to elicit strong antitumor immunity. Indeed, nPD-L1 substantially upregulated MHC-I expression in Hep3B cells (Figure 9, A–C). Moreover, increased IFNGR expression by nPD-L1 was also observed (Figure 9, D and E), and subsequently, nPD-L1 dramatically increased the expression of p-STAT1 in Hep3B cells compared with vector in response to IFN-γ, indicating the enhanced IFN-γ response of tumor cells (Figure 9F). Thus, enhanced MHC-I antigen presentation and IFN-γ signaling by nPD-L1 may unleash potent antitumor immunity.

## Discussion

The spatiotemporal organization of intracellular proteins is tightly regulated, which often determines cell fate (42). Proteins must function specifically at given times in certain cellular compartments (43). Cell surface PD-L1 in tumor cells either activates PD-1–mediated inhibitory signaling in T cells (5, 44, 45) or receives the signal from PD-1 to rapidly induce T cell killing resistance (46). In this study, we show that nuclear compartmentalization of PD-L1 mediates a protective epigenetic response to stimulus signals such as LA and overcomes intrinsic and acquired ICB resistance. Interestingly, nPD-L1 as a strong tumor suppressor exhibits the exact opposite function to that on the cell surface. It has been known that protein translocation usually leads to altered interactors and functions of the translocated protein (43); here, we propose that the anti- or protumor effect of PD-L1 depends on its subcellular distribution. Normally, PD-L1 acts as an inhibitory immune checkpoint on the cell membrane to suppress the activity of cytotoxic lymphocytes; nonetheless, in response to stimuli such as LA, PD-L1 protein translocates into the nucleus to activate genes of cellular senescence, JAK/STAT, and Hippo signaling pathways to suppress tumor malignancy and sensitize tumor cells to ICB. Thus, our study provides a new conceptual perspective on PD-L1 functionality.

One hundred thirty sites in more than 100 target proteins have been shown to be phosphorylated by AMPK, involving most branches of cell metabolism and function (47). AMPK was frequently reported to phosphorylate proteins to promote their nuclear translocation to activate gene expression in a genetic or epigenetic manner (31, 48–52). Blockage of nuclear translocation or inactivation of AMPK ameliorated cell death (53). AMPK facilitated nuclear translocation of PD-L1 and subsequently phosphorylated mH2A1 with the assistance of PD-L1 to epigenetically prevent tumorigenesis and boost antitumor immunity, providing new insights into the AMPK function in cancer.

Existing evidence, from both basic research and the clinical setting, that reveals histone variant mH2A1 as a tumor suppressor is compelling and convincing (12). In spite of the tangible function of mH2A1 in cell proliferation, tumorigenesis, stemness, senescence, and antitumor immunity, the molecular mechanism is unclear. Our study provides a clear pathway by which mH2A1 exerts its antitumor effect, which would favor the exploitation and clinical translation of new and improved therapeutic approaches that could induce accumulation of PD-L1 and p-AMPKα or phosphorylation of mH2A1-p146.

Nuclear PD-L1 translocation in tumor cells in hypoxic areas of tumor causes pyroptosis and thereby chronic tumor necrosis (8), which fuels tumor growth (54). However, we report in this study that induction of nuclear PD-L1 translocation inhibits tumorigenesis and enhances antitumor immunity. In general, chronic inflammation drives tumor growth while acute inflammation represses tumor growth (54). We speculate that LA-induced PD-L1 compartmentalization might produce acute inflammation beyond tumorigenesis suppression.

nPD-L1 suppressed tumor growth in antitumor immunity–dependent and –independent manners. Although nPD-L1 turned on 3 signaling pathways that may fuel tumor inhibition, senescence has been reported to regulate JAK/STAT and Hippo pathways (55–58). Not only does senescence per se impede tumor growth by cell cycle arrest and proliferation inhibition (59), but it also elicits antitumor immunity through upregulation of MHC expression and antigen presentation and enhancement of response to interferon (40, 41). Thus, we speculate that senescence may play a central role in nPD-L1–mediated tumor suppression, and the overall survival benefit seen in mice may be attributed to its capability of both decreasing tumor cell proliferation and increasing antitumor immunity.

Combination treatment of LA and ICB suppressed tumor growth in multiple tumor models, indicating a general antitumor effect of LA. Based on this study, tumor patients with high expression of PD-L1 are more suitable for LA treatment than others. Notably, elevated expression of PD-L1 was observed in tumor patients resistant to ICB therapy (60); thus, LA treatment may be a promising potential strategy for overcoming ICB resistance in cancers.

## Methods

### Sex as a biological variable.

For our animal study, sex was not considered as a biological variable. We used both male and female mice for our in vivo experiments.

### Cell lines.

Hep3B, CT26, and Hepa-1-6 cells were obtained from the American Type Culture Collection. Huh7, Tong, Mahlavu, and MHCC97H cells were from WheLab. Pan02 cells were from Shanghai Institute of Biochemistry and Cell Biology, Chinese Academy of Sciences. MHCC97H, Pan02, CT26, and Hepa-1-6 cells were maintained in Dulbecco’s modified Eagle medium containing 10% fetal bovine serum (FBS). Hep3B cells were cultured in MEM with 10% FBS. All cells were cultured at 37°C in a 5% CO_2_ incubator.

### Antibodies and reagents.

The following antibodies were used at 1:1,000 for immunoblotting: rabbit anti-AMPKα (clone D5A2; catalog 5831), rabbit anti-SAV1 (clone D6M6X; catalog 13301), rabbit anti-LATS2 (clone D83D6; catalog 5888), rabbit anti-GSK3B (clone 27C10; catalog 9315), rabbit anti-SIRT1 (clone D1D7; catalog 9475), rabbit anti-SMAD3 (catalog 9513), mouse anti–MHC-I heavy chain (clone EMR8-5; catalog 88274), rabbit anti-STAT1 (catalog 9172), rabbit anti–p-STAT1 (clone 58D6; catalog 9167), rabbit anti–p-AMPKα Thr172 (clone 40H9; catalog 2535), rabbit anti–PD-L1 (clone E1L3N; catalog 13684), and rabbit anti–lamin B1 (clone D4Q4Z; catalog 12586) from Cell Signaling Technology; rabbit anti-SMAD7 (polyclonal; catalog SAB4200345), rabbit anti–MHC-I–β2M (polyclonal; catalog SAB2105109), mouse anti-tubulin (clone B-5-1-2; catalog T5168), and mouse anti-FLAG (clone M2; catalog F3165) from Sigma-Aldrich; mouse anti-HA (clone 16B12; catalog 901502) from BioLegend; rabbit anti-NKD1 (polyclonal; catalog H00085407-D01P), rabbit anti-NFATC1 (polyclonal; catalog PA5-79730), mouse anti-FOXO1 (clone 3B6; catalog MA5-17078), rabbit anti-GADD45B (polyclonal; catalog PA5-43160), mouse anti-IFNLR1 (clone 601106; catalog MA5-24271), mouse anti-CREBBP (clone 2B6; catalog H00001387-M02), rabbit anti–IL-21R (polyclonal; catalog PA5-19982), mouse anti-CDKN1A (clone OTI4B11; catalog CF808276), rabbit anti-IFNGR (polyclonal; catalog 10808-1-AP), mouse anti-mH2A1 (clone CL5245; catalog MA5-31412), and mouse anti-myc (clone 9E10; catalog MA1-980) from Thermo Fisher Scientific; and rabbit anti–thiophosphate ester (clone 51-8; catalog ab92570) from Abcam. The following antibodies were used at 1:100 for immunoprecipitation: rabbit anti–PD-L1 (clone E1L3N; catalog 13684) and rabbit anti–p-AMPKα Thr172 (clone 40H9; catalog 2535) from Cell Signaling Technology; and mouse anti-HA (clone 16B12; catalog 901502) from BioLegend. The following antibodies were used at 1:200 for immunofluorescence and Duolink assays: mouse anti–PD-L1 (clone OTI2C7; catalog LS-C338364) from LifeSpan BioSciences; and rabbit anti–p-AMPKα Thr172 (clone 40H9; catalog 2535) from Cell Signaling Technology. The following antibodies were used at 1:100 for flow cytometry: mouse anti–MHC-I heavy chain (clone EMR8-5; catalog 88274) from Cell Signaling Technology; and rabbit anti-IFNGR (polyclonal; catalog 10808-1-AP) from Thermo Fisher Scientific.

Metformin (catalog S5958), A-769662 (catalog S2697), AICAR (catalog S1802), compound C (catalog S7840), α-lipoic acid (LA) (catalog S3998), and ivermectin (catalog S1351) were purchased from Selleckchem, Matrigel from BD Biosciences, and IFN-γ protein (catalog IF002) from Sigma-Aldrich.

### Generation of stable transfectants.

Endogenous PD-L1–knockout and nPD-L1–overexpressed stable cells were constructed as described previously (8). Briefly, we mutated _249_LCL_251_ to ACA in PD-L1 sequence and cloned this mutated *PD-L1* gene (nPD-L1) to a pCDH-CMV-Puro vector. Then this construct was delivered into endogenous PD-L1–knockout cells via lentivirus infection. To generate CRISPR-mediated AMPKα-knockout Hep3B and Hepa-1-6 cells, sgRNA sequences were used and subcloned into pLenti-CRISPR V2 GFP vector (Addgene) as described previously (9). To package lentivirus, the constructs combined with 2 packaging plasmids were cotransfected into HEK293T cells. The medium was changed at 24 hours after transfection, and the viral particles were collected at 48 hours and 72 hours after transfection. Cells were infected with viral particles in the presence of Polybrene (10 μg/mL) overnight. Then puromycin (2 μg/mL) or G418 (2,000 μg/mL) was used to select infected cells.

### Duolink assay and immunofluorescence.

Hep3B cells were seeded on 4-well chamber slides and treated with LA (1 mM), the AMPKα activator AICAR (500 μM), the importin α/β inhibitor ivermectin (25 μM), or the p-AMPKα inhibitor compound C. Then Duolink assays were performed with a Duolink In Situ Red Starter Kit (DUO92101, Sigma-Aldrich) according to the manufacturer’s instructions. The interactions between PD-L1 and p-AMPKα were analyzed and displayed as distinct red spots. The immunofluorescence was performed as described previously (8).

### Cellular fractionation.

Cellular fractionation was performed as described previously (8). Briefly, cells were washed with ice-cold PBS and harvested by lysis in Nori buffer (made in-house with 10 mM KCl; 2 mM MgCl_2_; 20 mM HEPES, pH 7.0; 0.5% NP-40; 1 mM PSMF; 2 μg/mL aprotinin; 1 mM Na_3_VO_4_; and 10 mM NaF), and then incubated on ice for 15 minutes. Cell lysate was homogenized for 60 strokes in a Dounce homogenizer (Wheaton). The homogenate was centrifuged at 1,600*g* for 5 minutes at 4°C to sediment the nuclei. The supernatant was centrifuged at 17,000*g* for 30 minutes at 4°C, and the resulting supernatant formed the non-nuclear fraction. The nuclear pellet was washed with lysis buffer and then resuspended in NETN buffer (made in-house with 1 mM EDTA; 150 mM NaCl; 20 mM Tris-HCl, pH 8.0; 1 mM Na_3_VO_4_; 10 mM NaF; 1 mM PMSF; 0.5% NP-40; and 2 μg/mL aprotinin) and subsequently sonicated for 15 cycles at 20 seconds per cycle. The supernatant was collected for nuclear fraction with centrifugation at 17,000*g* for 30 minutes at 4°C.

### Immunoblotting and immunoprecipitation.

Immunoblotting and immunoprecipitation were performed as described previously (61). For immunoblotting, cell lysate with loading buffer was incubated in boiled water for 10 minutes and then subjected to SDS-PAGE (10%). Proteins were transferred to a PVDF membrane. After being blocked with skimmed milk (5%) for 1 hour at room temperature, the part of the PVDF membrane with target protein was incubated in 5% BSA with primary antibody overnight. The PVDF membrane was washed 3 times with PBST (PBS plus 0.1% Tween-20) and then incubated with secondary antibody for 2 hours at room temperature. After being washed with PBST, the PVDF membrane with target proteins was detected using the ECL system. For immunoprecipitation, 2 μg of the relevant antibody was added to cell lysate and incubated overnight at 4°C. Cell lysate was then immunoprecipitated with protein G–Sepharose beads for 3 hours at 4°C. Immunoprecipitates were then subjected to immunoblotting analysis.

### 3D cell culture.

A thin layer of Matrigel was spread to each well of a prechilled 24-well plate and incubated at 37°C for 30 minutes to allow the Matrigel to solidify. Then Hep3B cells were trypsinized into single cells and resuspended in corresponding medium containing 10% Matrigel. Cell suspensions were pipetted onto the gel surface and were allowed to attach to Matrigel. Fresh medium containing 10% Matrigel was changed every 2 days. After 10 days of culture, photomicrographs of representative colonies were taken. Colonies were then stained with crystal violet, and colonies larger than 100 μm were counted.

### Sphere-forming assay.

Cells were dissociated into single cells and plated in 6-well ultra-low-attachment plates at a density of 10,000 viable cells per well. After 10 days of culture, spheres larger than 50 μm were counted.

### In vitro kinase assays.

In vitro kinase assays were performed as described previously (10, 62). Briefly, recombinant myc–PD-L1, HA-AMPKα, and FLAG-mH2A1 proteins purified from *E.* *coli* were subjected to kinase assays in a solution consisting of HEPES–Brij buffer, 0.3 mM AMP, and 0.2 mM ATP (with 0.5 mCi/mL γ-^32^P-ATP for radioactive assay) for 30 minutes at 30°C. The kinase reactions were stopped with SDS sample loading buffer and by heating at 100°C for 10 minutes. Samples were then subjected to SDS-PAGE, Coomassie blue staining, and autoradiography.

### Quantitative reverse transcription PCR.

Total RNA was isolated from Hep3B-PD-L1-KO-vector and Hep3B-PD-L1- KO-nPD-L1 cells using a TRIzol Plus RNA Purification Kit (Invitrogen). cDNA was synthesized using an iScript cDNA Synthesis Kit (Bio-Rad). Quantitative PCR was performed with iQ SYBR Green Supermix (Bio-Rad). Primers for quantitative PCR are shown in Table 3.

### RNA sequencing and bioinformatics analysis.

Total RNA from Hep3B-PD-L1-KO-vector and Hep3B-PD-L1-KO-nPD-L1 cells was isolated using a TRIzol Plus RNA Purification Kit (Invitrogen). RNA sequencing and analysis were performed by Novogene Co. Ltd.

### Antibody generation and detection.

An anti–mH2A1 S146 phosphorylation antibody (anti–mH2A1-p146) was produced against the region near the Ser146 phosphorylation site of mH2A1.

The phosphorylated synthetic peptide [C-KSQKKPVS(p)KKAGGKK] was used for immunization in the mice, which were purchased from Shanghai SLAC Laboratory Animals Co. Ltd. The antibody was produced as described previously (45). For detecting mH2A1 S146 phosphorylation, antibodies were preincubated with cold or hot peptide for 2 hours at 4°C and applied to pull-down of S146 phosphorylation of mH2A1 by immunoprecipitation as described above.

### Animal studies.

For tumorigenesis in vivo, Hep3B cells mixed with Matrigel (1:1, vol/vol) were subcutaneously injected into the flanks of nude mice. Two weeks after implantation, tumor incidence was analyzed. For nPD-L1–enriched Hep3B tumor growth, 4 × 10^6^ cells in 100 μL of sterile PBS mixed with 100 μL of Matrigel were subcutaneously injected into the flanks of nude mice. For growth of nPD-L1–knockin Hep3B/Huh7/Mahlavu tumors, 4 × 10^6^ cells in 100 μL of sterile PBS mixed with 100 μL of Matrigel were subcutaneously injected into the flanks of nude mice. For CT26 tumors, 1 × 10^6^ cells mixed with Matrigel (1:1, vol/vol) were subcutaneously injected into the flanks of BALB/c mice. For Pan02 and Hepa-1-6 tumors, 1 × 10^6^ cells were subcutaneously injected into the flanks of 6-week-old female C57BL/6 mice.

For the in vivo anti–PD-1–resistant model, 1 × 10^6^ Hepa-1-6 cells were subcutaneously injected into the flanks of 6-week-old female C57BL/6 mice. PD-1 mAb was injected intraperitoneally 4 times at 3-day intervals. After tumor volumes reached the endpoint size, 1,500 mm^3^, tumors were excised, minced into small pieces, and digested in collagenase (Sigma-Aldrich) and DNase I (Sigma-Aldrich). Then cell suspensions were filtered with 70-μm strainers, washed twice with PBS, and cultured in completed medium. Cells from Hepa-1-6 tumors that did not respond to PD-1 mAb treatment were implanted into immunocompetent mice to generate the intrinsic resistance model of ICB, while cells from tumors that initially responded but eventually acquired resistance to PD-1 mAb treatment were used to generate the acquired resistance model.

For LA treatment, LA (100 mg/kg/d) was orally administered to mice in drinking water. For A-769662 treatment, mice were treated (i.p.) with A-769662 (30 mg/kg) every other day. For PD-1 antibody treatment, mice were treated with 100 μg/dose of PD-1 antibody (BE0146, Bio X Cell) twice a week.

All animal experiments were performed under guidelines approved by the Animal Care and Use Committees of the Laboratory Animal Research Center at Xiangya Medical School of Central South University. All mice were obtained from Shanghai SLAC Laboratory Animals Co. Ltd. and maintained in the standard housing conditions (temperatures of 18°C–24°C with 40%–60% humidity and a 14-hour light/10-hour dark cycle) recommended by The Jackson Laboratory.

### FACS analysis of CD8^+^ TILs.

Excised tumors were digested in collagenase (Sigma-Aldrich) and DNase I (Sigma-Aldrich). Cell suspensions were filtered with 70-μm strainers, and the recovered cells were washed twice with PBS. Percoll gradient centrifugation was then performed to isolate lymphocytes. T cells were stained with CD8 (clone 53-6.7), IFN-γ (clone XMG1.2), and GZMB (clone QA16A02), and the stained samples were analyzed by a BD FACSCanto II cytometer (BD Biosciences).

### Statistics.

GraphPad Prism 9.0 was used for statistical analysis. Quantitative data are shown as mean ± SD. A log-rank test was used for survival analysis. Normality and equal variances between sample groups were assessed by Shapiro-Wilk and Brown-Forsythe tests, respectively. When normality and equal variance were achieved between sample groups, 1-way ANOVA (followed by Dunnett’s correction) or unpaired 2-tailed *t* test was used. When normality was achieved but equal variance failed, Brown-Forsythe 1-way ANOVA (followed by Dunnett’s T3 correction) or unpaired 2-tailed *t* test with Welch’s correction was performed. When normality and equal variance of sample groups failed, Kruskal-Wallis 1-way ANOVA (followed by Dunn’s correction) or Mann-Whitney tests were performed. Differences were considered statistically significant when *P* was less than 0.05.

### Study approval.

Animal work was conducted in accordance with the *Guide for the Care and Use of Laboratory Animals* (Ministry of Health, China). The protocol of animal work was reviewed and approved by the Animal Care and Use Committees of the Laboratory Animal Research Center at Xiangya Medical School of Central South University.

### Data availability.

Raw RNA sequencing data have been deposited to the NCBI’s Gene Expression Omnibus (GEO) database with accession number GSE276400. Values for all data points shown in graphs are provided in the Supporting Data Values file.

## Author contributions

MCH and JH designed and conceived the study. JH, XZ, JZ, and MCH wrote the manuscript. JH, YL, ZY, RM, and SW performed experiments and analyzed data. ZY performed RNA sequencing and bioinformatics analysis. SW drew the schematic model and performed statistical analysis. CWMC analyzed the sequence and structure of PD-L1. MCH and JH supervised the entire project.

## Supplementary Material

Supplemental data

Unedited blot and gel images

Supplemental table 1

Supplemental table 2

Supporting data values

## Figures and Tables

**Table 3:**
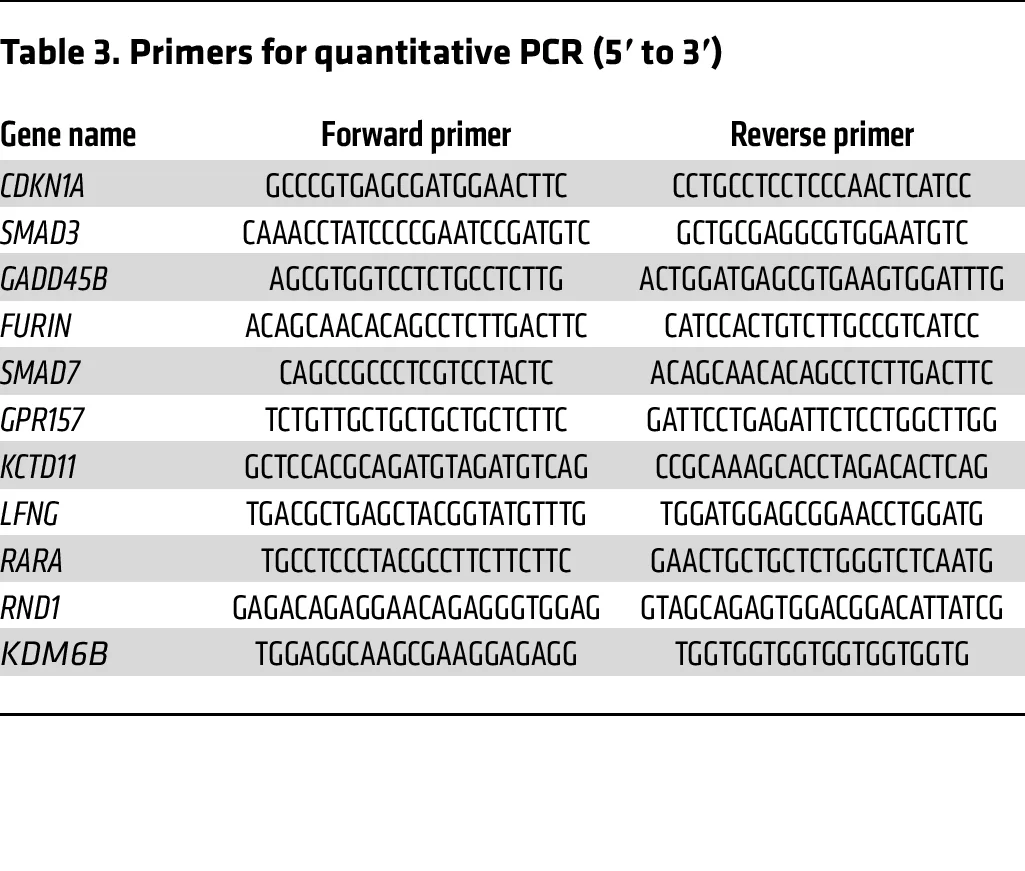
Primers for quantitative PCR (5′ to 3′)

**Table 2:**
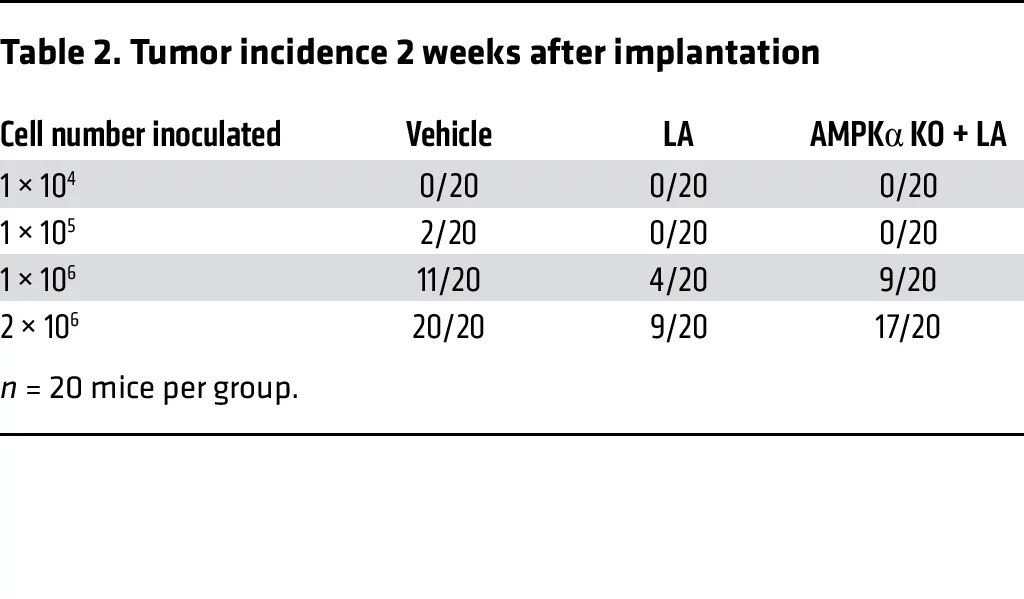
Tumor incidence 2 weeks after implantation

**Table 1:**
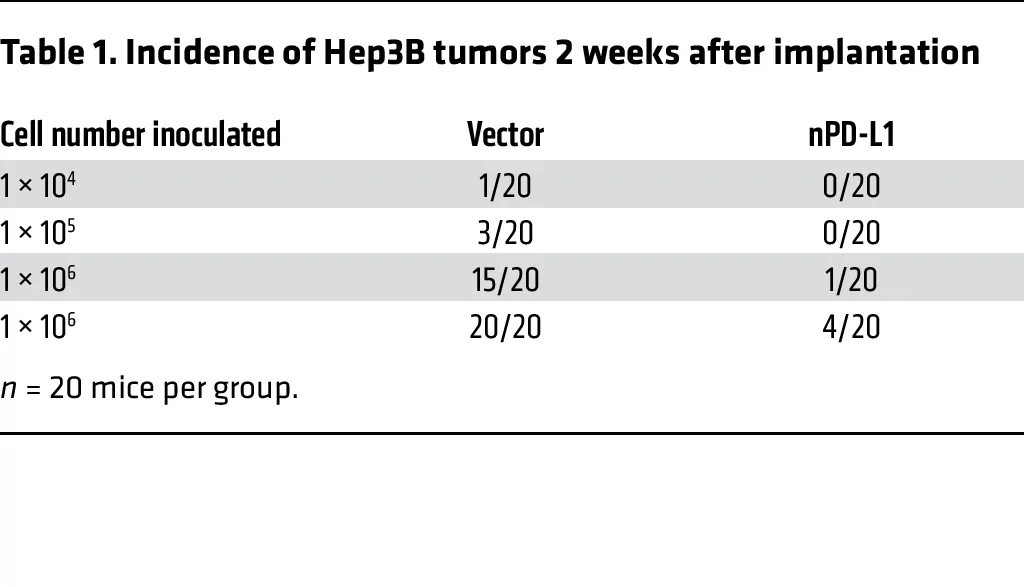
Incidence of Hep3B tumors 2 weeks after implantation

## Version 1 11/15/2024

Electronic publication

## Version 2 10/26/2026

Corrections to supporting data values file due to copy and paste errors relating to Figure 1, F & I, Figure 8E, and Supplemental Figure 11B.
