# Supplementary material for: Nuclear PD-L1 compartmentalization suppresses tumorigenesis and overcomes immunocheckpoint therapy resistance in mice via histone macroH2A1
Source: J Clin Invest. 2024 Nov 15;134(22):e181314. doi: 10.1172/JCI181314 (PMC11563670; doi:10.1172/JCI181314)
Supplement: Supplemental data [file jci-134-181314-s145.pdf]

Supplementary Information

Nuclear PD-L1 compartmentalization suppresses tumorigenesis and overcomes immunocheckpoint therapy resistance in mice via histone macroH2A1

Yong Liu, Zhi Yang, Shuanglian Wang, Rui Miao, Chiung-Wen Mary Chang, Jingyu Zhang, Xin Zhang, Mien-Chie Hung, Junwei Hou

Supplemental Figure 1

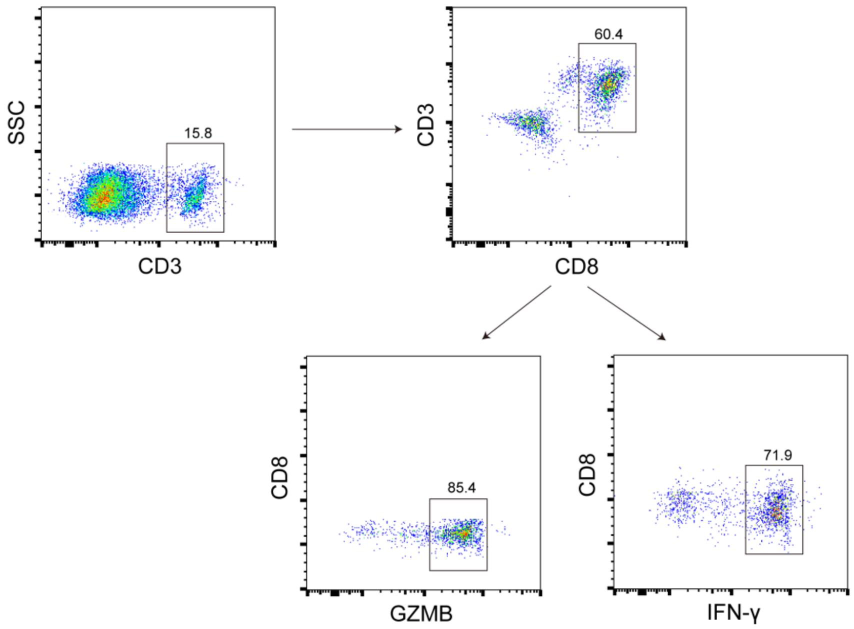

Supplemental Figure 1 The flow cytometry gating strategy for Figure 2, E and F.

Supplemental Figure 2

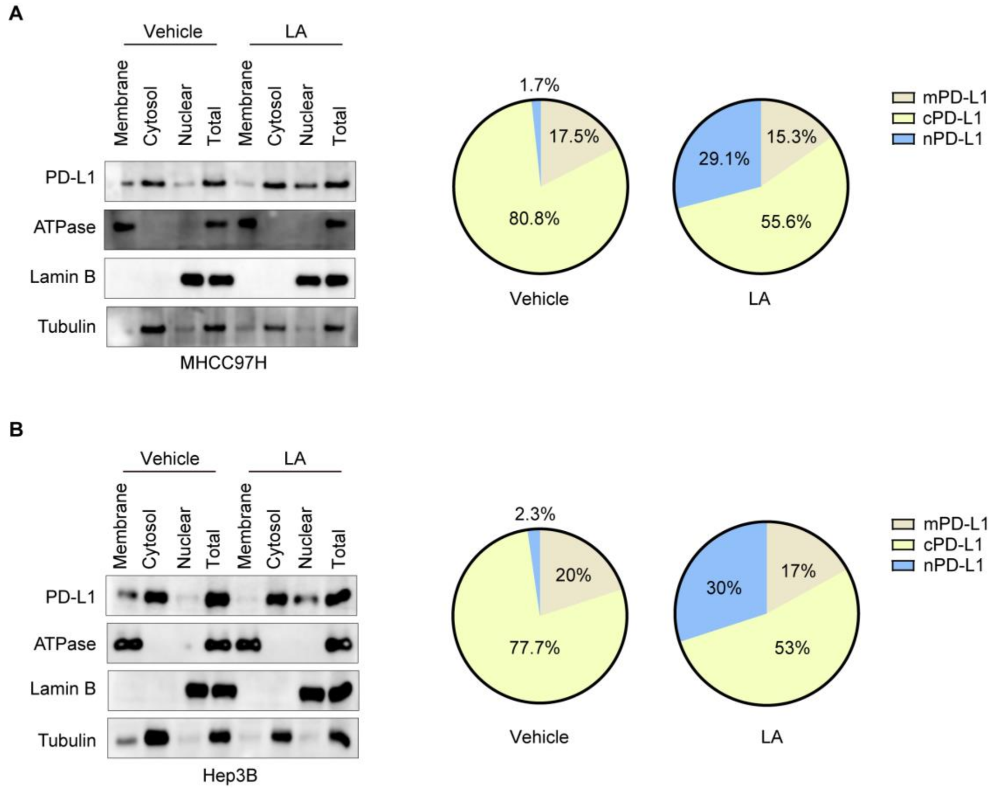

Supplemental Figure 2 Cellular fractionation analysis of PD-L1 in MHCC97H and Hep3B cells treated with LA. (**A**) Left: Immunoblotting of PD-L1 in MHCC97H cells. Right: The ratio of PD-L1 expression in membrane, cytosol, and nucleus according to the immunoblotting results in Left. mPD-L1, membrane PD-L1; cPD-L1, cytosol PD-L1; nPD-L1, nuclear PD-L1. (**B**) Left: Immunoblotting of PD-L1 in Hep3B cells. Right: The ratio of PD-L1 expression in membrane, cytosol, and nucleus according to the immunoblotting results in Left. mPD-L1, membrane PD-L1; cPD-L1, cytosol PD-L1; nPD-L1, nuclear PD-L1.

Supplemental Figure 3

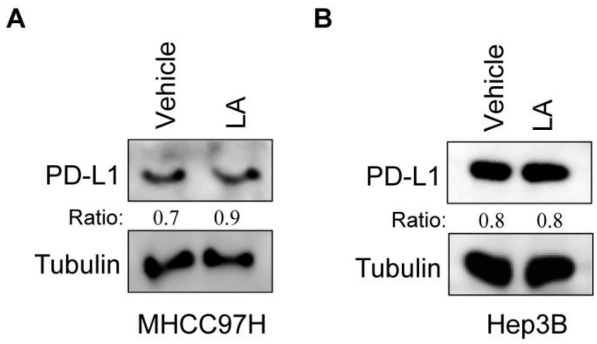

Supplemental Figure 3 Immunoblotting of PD-L1 in MHCC97H and Hep3B cells treated with LA (200 μM) for 48 hours.

Supplemental Figure 4

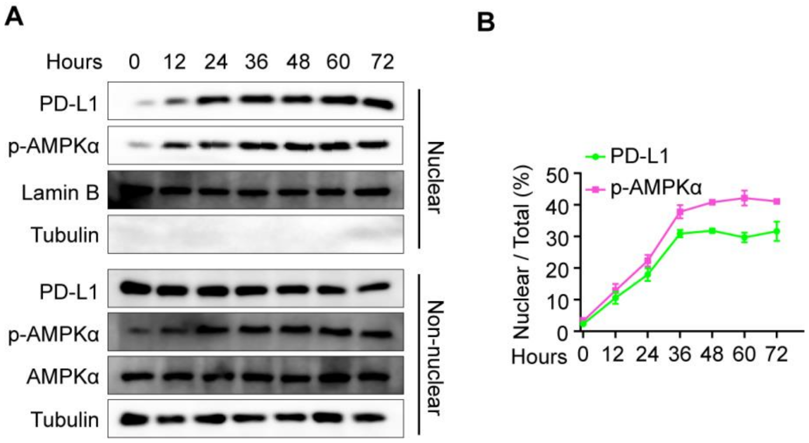

Supplemental Figure 4 Time course analysis of LA-induced nuclear PD-L1 translocation. (**A**) Immunoblotting of PD-L1 and p-AMPKα in cellular fractions at the indicated time points in Hep3B cells treated with LA (200 μM). The experiment was repeated three times with similar results. (**B**) Kinetics of

nuclear PD-L1 and p-AMPKα translocation (n=3). Diagram showing the relative ratio of nuclear PD-L1 to total PD-L1 or nuclear p-AMPKα to total p-AMPKα based on immunoblot quantification in **A** using Image J software. Data shown are representative of three independent experiments.

Supplemental Figure 5

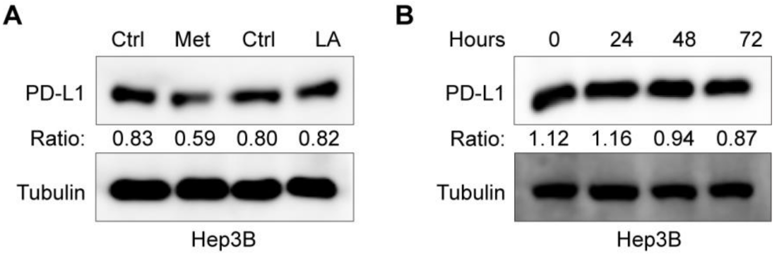

Supplemental Figure 5 Impact of LA treatment on PD-L1 expression. (**A**) Immunoblotting of PD-L1 in Hep3B cells treated with metformin (Met) (5 mM) or LA (200 μM) for 24 hours. (**B**) Immunoblotting analysis of PD-L1 expression at the indicated time points in Hep3B cells treated with LA (200 μM).

Supplemental Figure 6

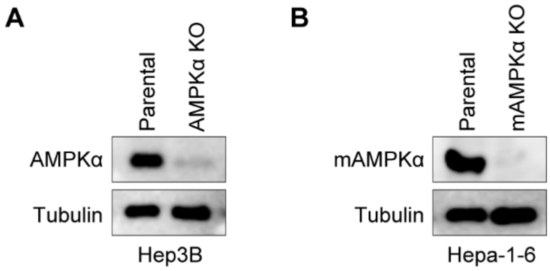

Supplemental Figure 6 Deletion of AMPKα in Hep3B and Hepa-1-6 cells. (**A**) Knockout (KO) of AMPKα in Hep3B cells. (**B**) KO of mouse AMPKα

(mAMPKα) in Hepa-1-6 cells.

Supplemental Figure 7

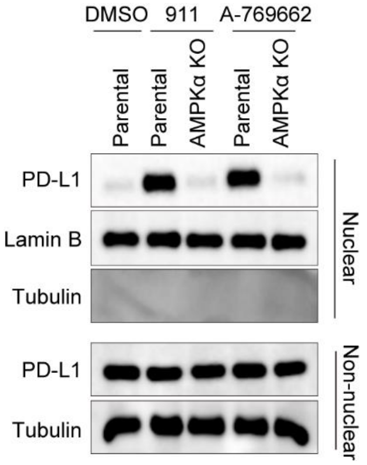

Supplemental Figure 7 Detection of PD-L1 expression in nucleus by cellular fractionation and immunoblotting in Hep3B parental or AMPKα-knockout (KO) cells treated with compound 911 (10 μM) or A-769662 (2 μM).

Supplemental Figure 8

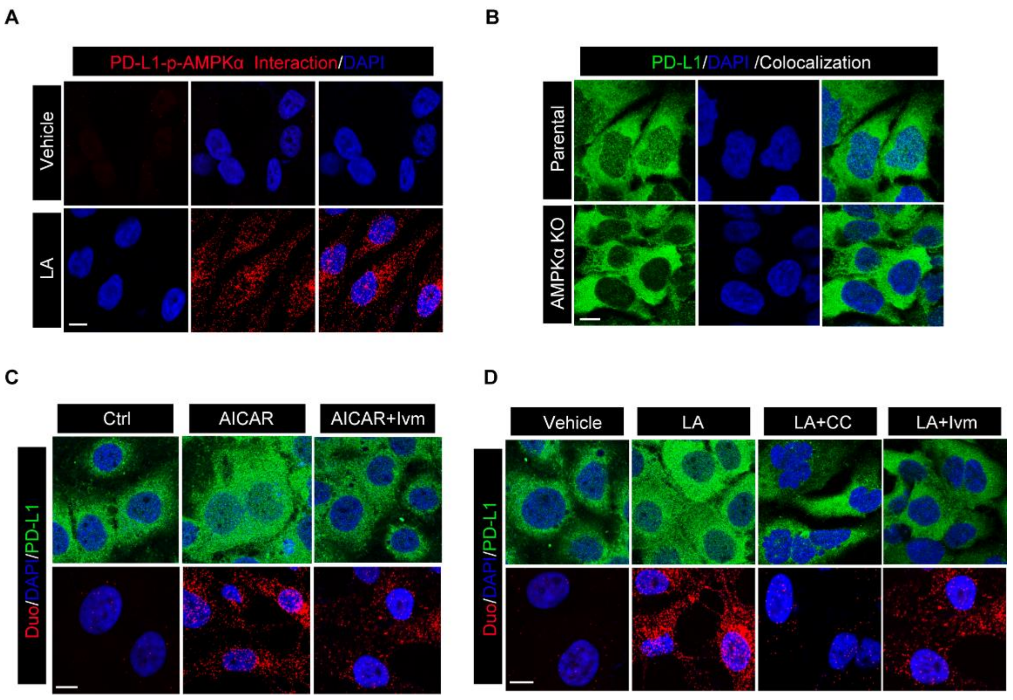

Supplemental Figure 8 LA-induced nuclear PD-L1 was associated with p-AMPKα. (**A**) Duolink assay (red dot: interaction between p-AMPKα and PD-L1) with antibodies specific for p-AMPKα and PD-L1 in Mahlavu cells treated with LA. Scale bar, 20 μm. (**B**) Confocal microscopy analysis of PD-L1 expression in LA-treated Mahlavu cells with deletion of AMPKα. Scale bar, 20 μm. (**C**) Mahlavu cells were treated with AMPKα activator AICAR (500 μM) or importin α/β inhibitor ivermectin (25 μM). Localization of PD-L1 or PD-L1–p-AMPKα interaction (red dots) was analyzed by confocal microscopy (upper) and Duolink assay (bottom). Scale bar, 20 μm. (**D**) Mahlavu cells were treated with LA, p-AMPKα inhibitor compound C (CC), or ivermectin. Localization of PD-L1 or PD-L1–p-AMPKα interaction (red dots) was analyzed by confocal microscopy (upper) and Duolink assay (bottom). Scale bar, 20 μm.

Supplemental Figure 9

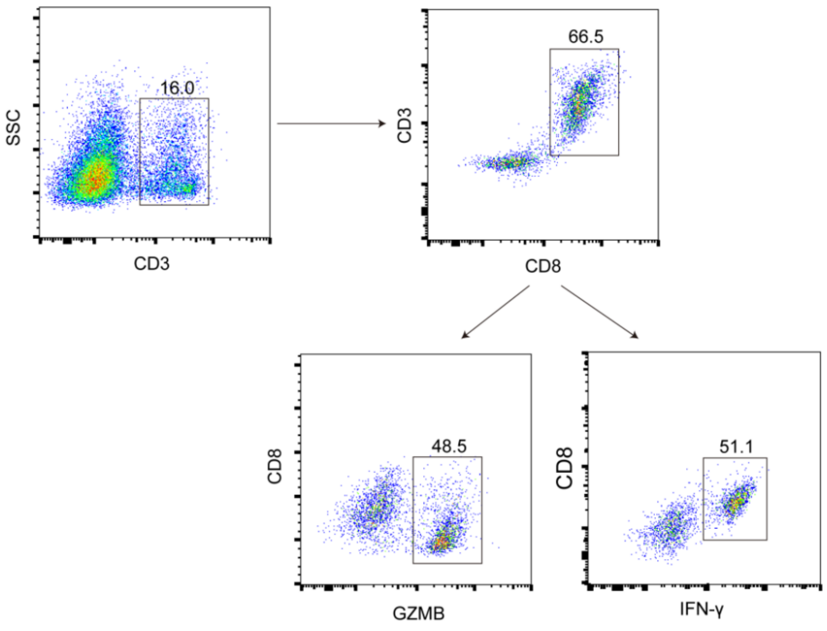

Supplemental Figure 9 The flow cytometry gating strategy for Figure 5, G and H.

Supplemental Figure 10

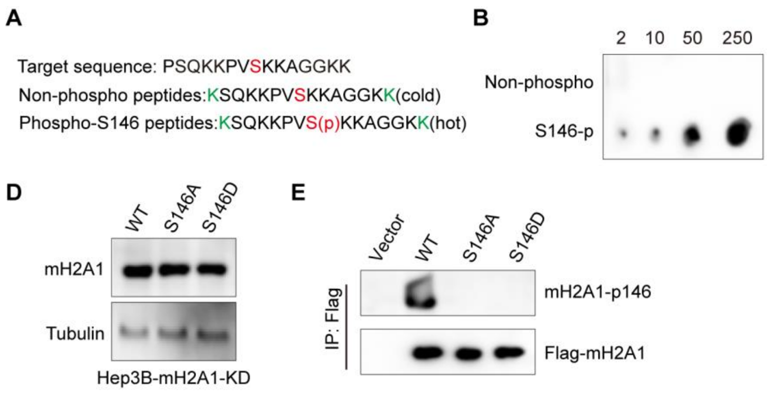

Supplemental Figure 10 Generation and validation of the anti-macroH2A1-p146 antibody. (**A**) Protein sequence of synthesized macroH2A1 (mH2A1) peptides for generating the anti-mH2A1-p146 antibody. (**B**) Titration of the indicated mH2A1 peptides with or without S146 phosphorylation to show that the developed anti-mH2A1-p146 antibody

specifically recognized the S146-p epitope in the dot blot analysis. (**C**) Knockdown (KD) of endogenous mH2A1 in Hep3B cells. (**D**) Enforced expression of wild-type (WT) or mutated (S146A and S146D) mH2A1 in stable cells established in (**C**). (**E**) IP analysis showed that S146A/S146D mutations abolished the recognition of mH2A1-p146 by the generated antibody.

Supplemental Figure 11

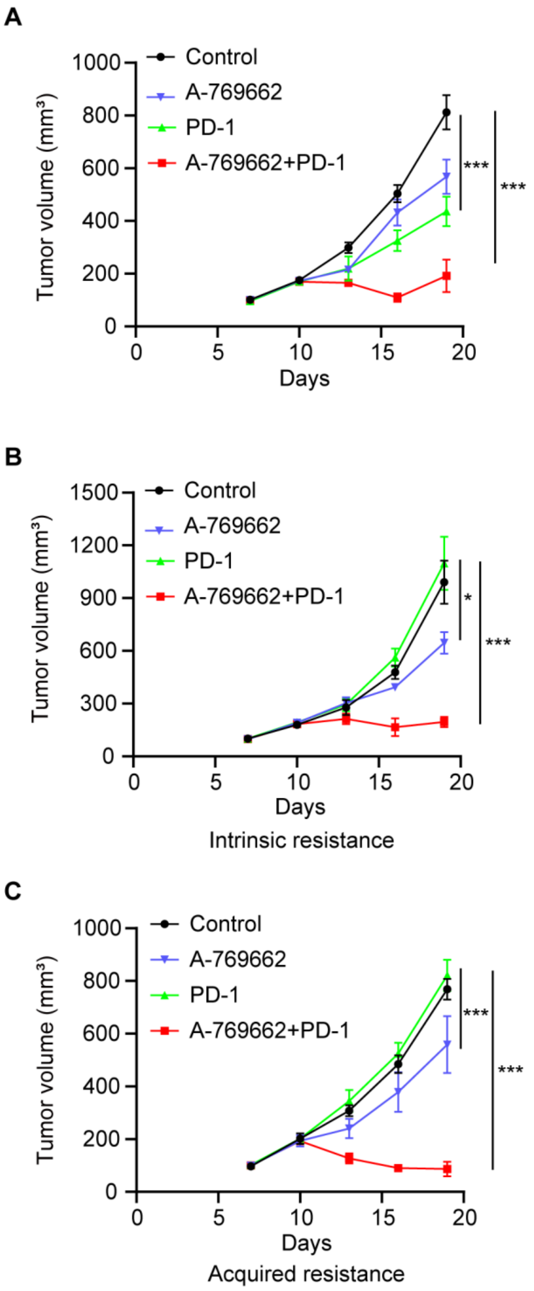

Supplemental Figure 11 A-769662 overcame the intrinsic and acquired resistance to ICB. Immunocompetent C57BL/6 mice bearing Hepa-1-6 tumors were administrated via i.p. with A-769662 (30 mg/kg) or PD-1 antibody alone, or in combination. (**A**) Tumor growth of Hepa-1-6 parental tumors (n = 6 mice/group). (**B**) Tumor growth of Hepa-1-6 tumors with intrinsic resistance to PD-1 antibody (n = 10 mice/group). (**C**) Tumor growth of Hepa-1-6 tumors with acquired resistance to PD-1 antibody (n = 10 mice/group). Data shown are mean + SD. 1-way ANOVA (Dunnett's correction) for (**A**). Kruskal-Wallis 1-way

ANOVA (Dunn's correction) for (**B**). Brown-Forsythe 1-way ANOVA (Dunnett T3 correction) for (**C**). $*p < 0.05$, $***p < 0.001$.
