# Supplementary material for: Nuclear PD-L1 compartmentalization suppresses tumorigenesis and overcomes immunocheckpoint therapy resistance in mice via histone macroH2A1
Source: J Clin Invest. 2024 Nov 15;134(22):e181314. doi: 10.1172/JCI181314 (PMC11563670; doi:10.1172/JCI181314)

The uncropped/unedited versions of all gel and blot images

Full unedited gel for Fig. 1A

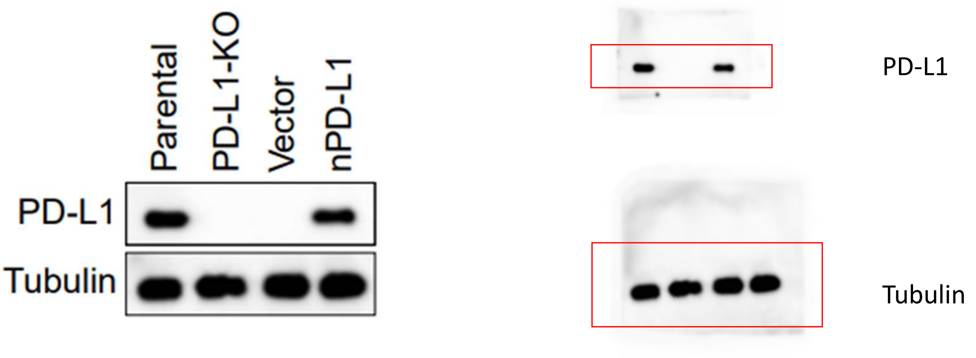

Full unedited gel for Fig. 1B

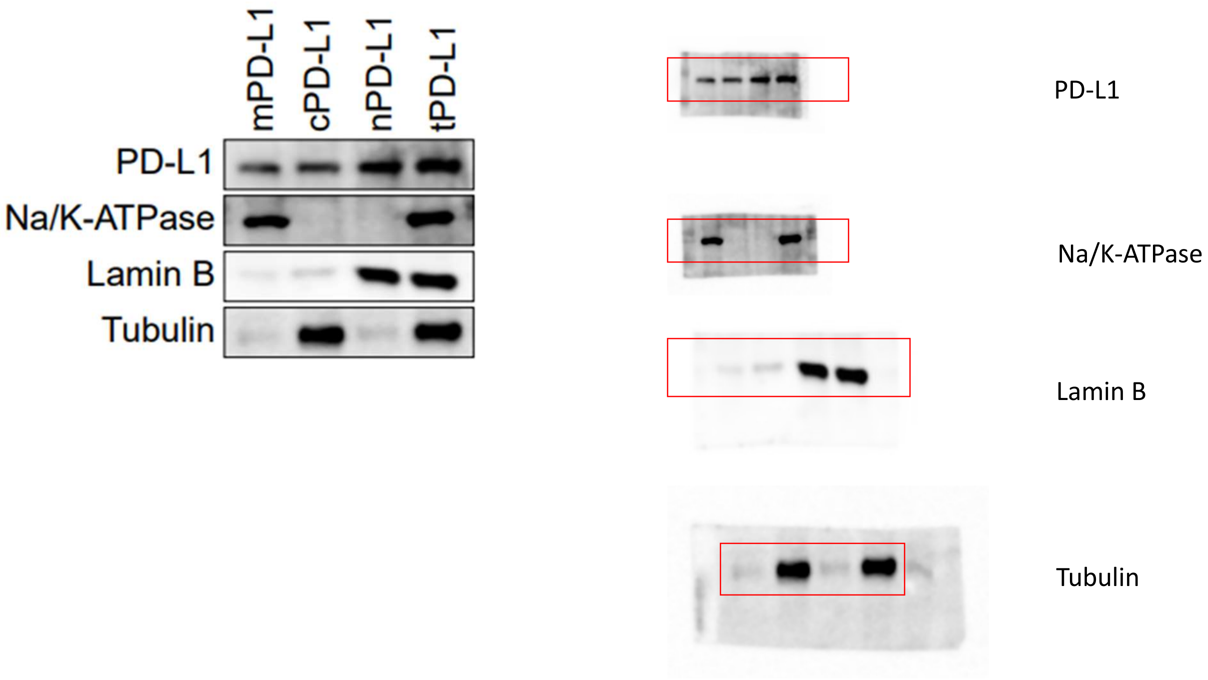

Full unedited gel for Fig. 1G

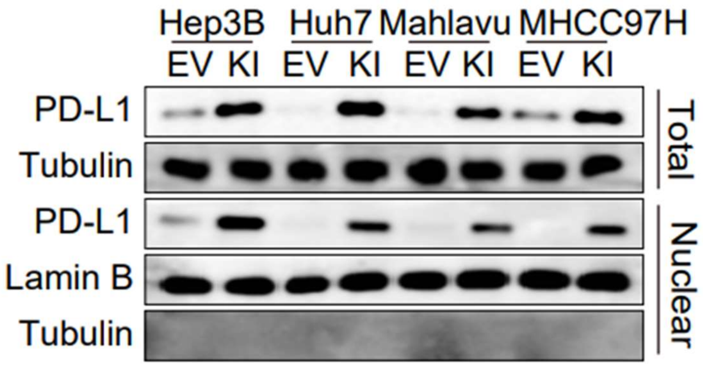

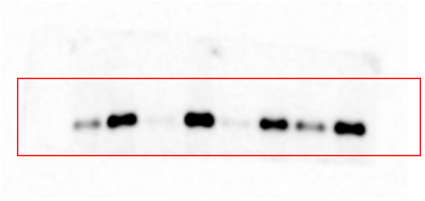

PD-L1(Total)

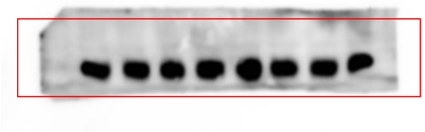

Tubulin(Total)

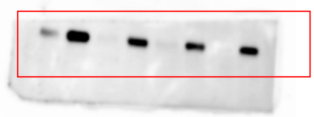

PD-L1(Nuclear)

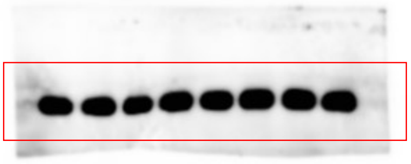

Lamin B(Nuclear)

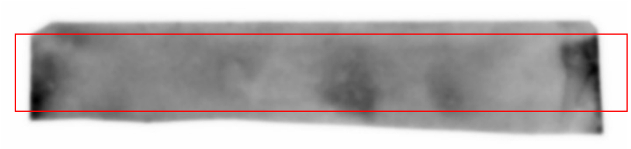

Tubulin(Nuclear)

Full unedited gel for Fig. 2B

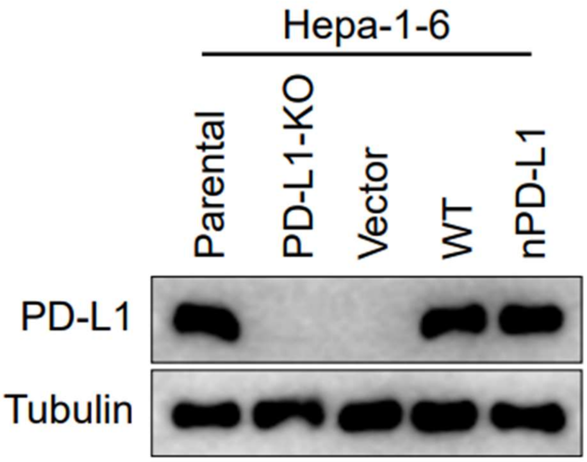

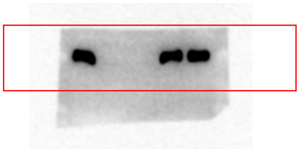

PD-L1

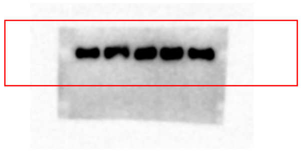

Tubulin

Full unedited gel for Fig. 3B

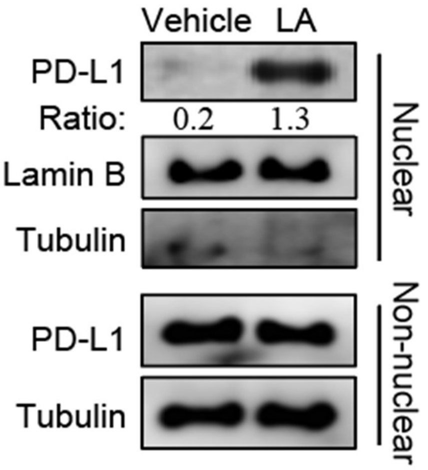

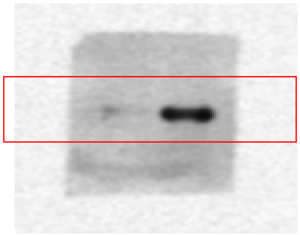

PD-L1(Nuclear)

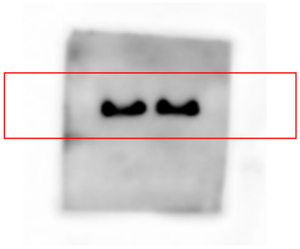

Lamin B(Nuclear)

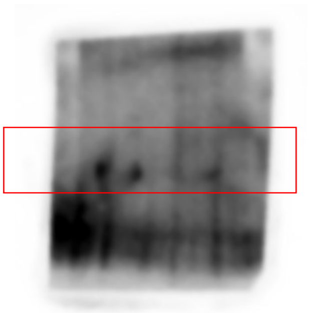

Tubulin(Nuclear)

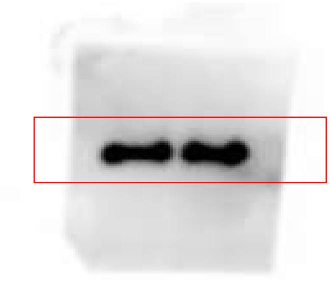

Tubulin(Non-nuclear)

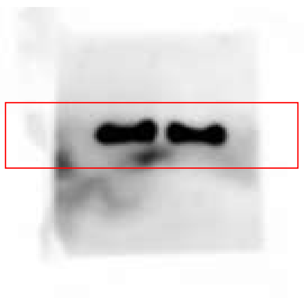

PD-L1(Non-nuclear)

Full unedited gel for Fig. 3D

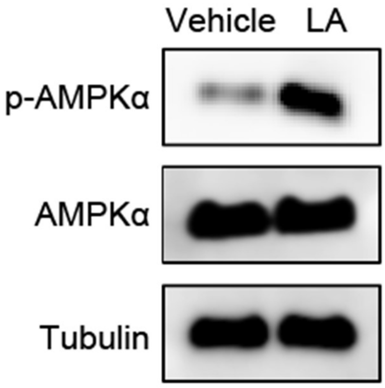

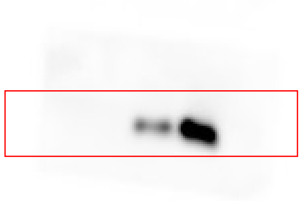

p-AMPKα

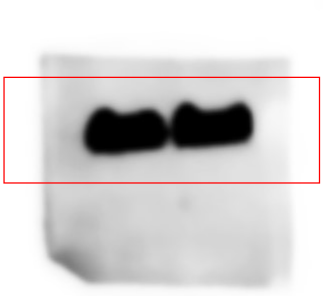

AMPKα

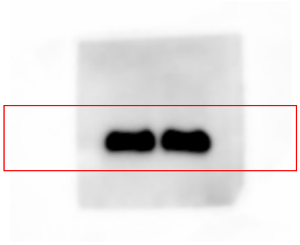

Tubulin

Full unedited gel for Fig. 3F

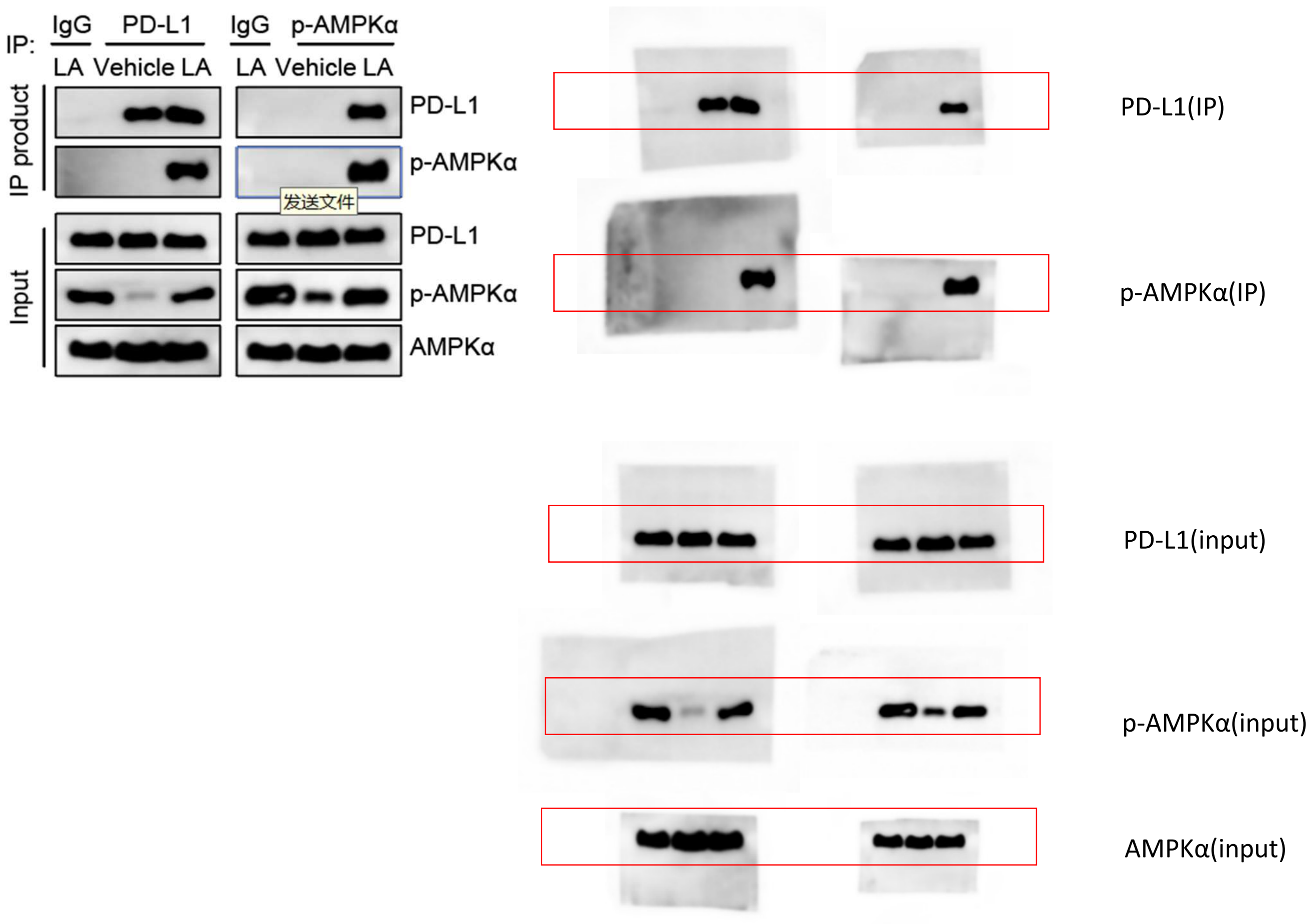

Full unedited gel for Fig. 3I

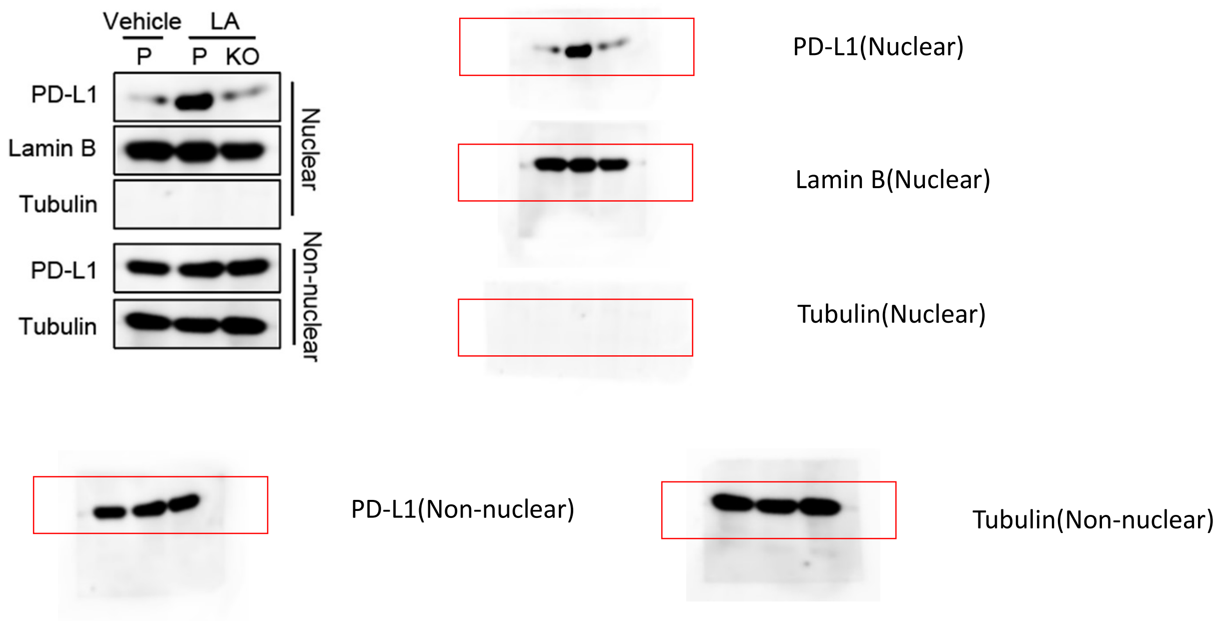

Full unedited gel for Fig. 6K

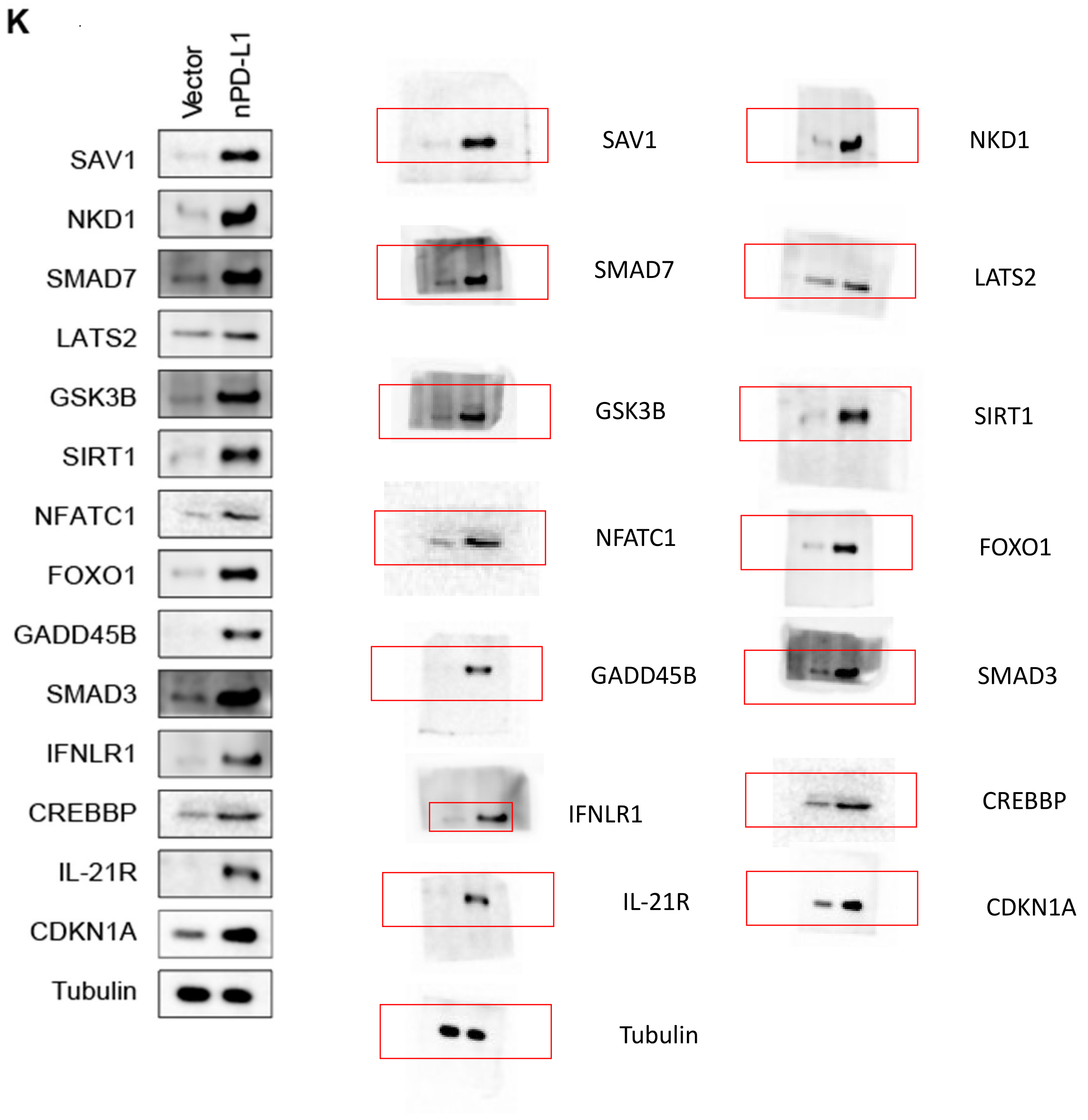

Full unedited gel for Fig. 7A

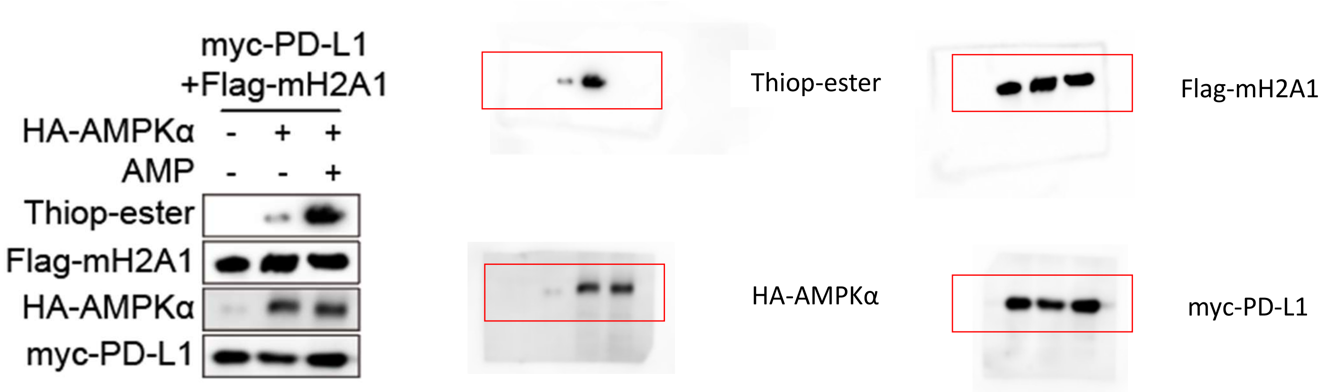

Full unedited gel for Fig. 7C

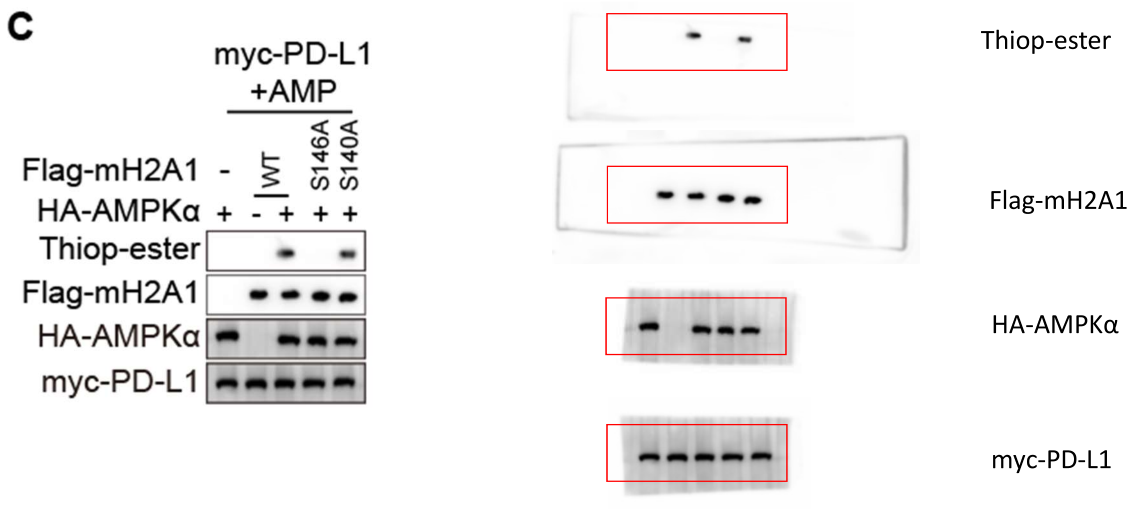

Full unedited gel for Fig. 7D

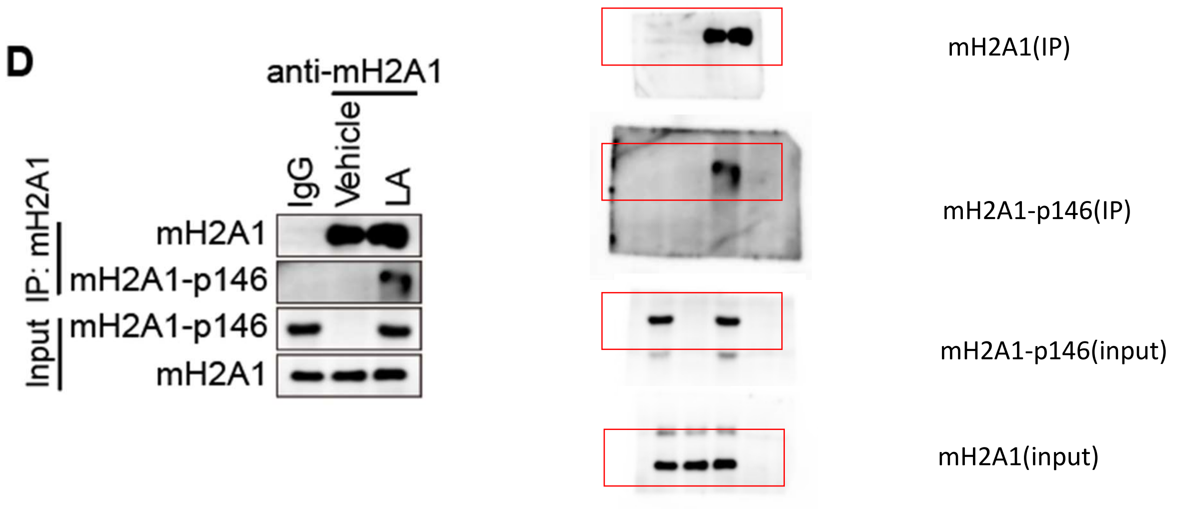

Full unedited gel for Fig. 7E

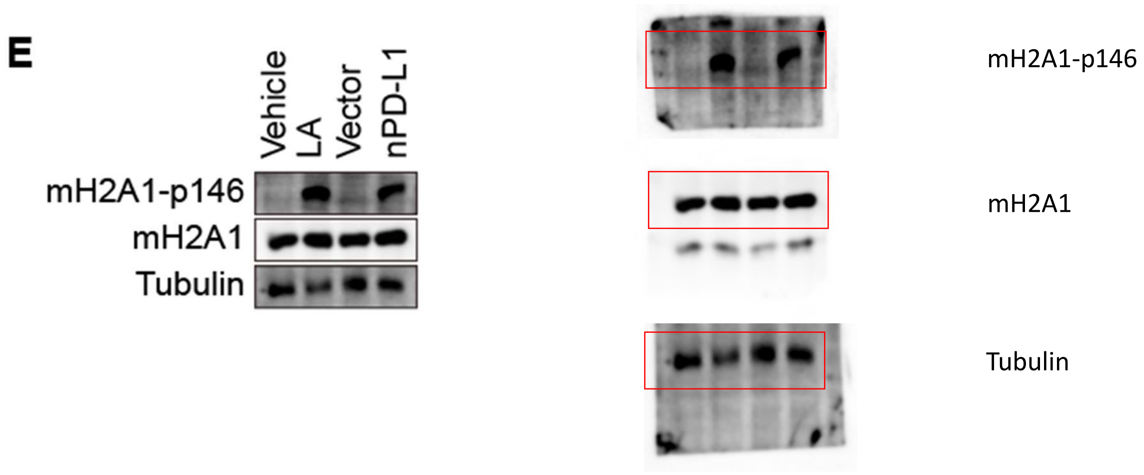

Full unedited gel for Fig. 7F

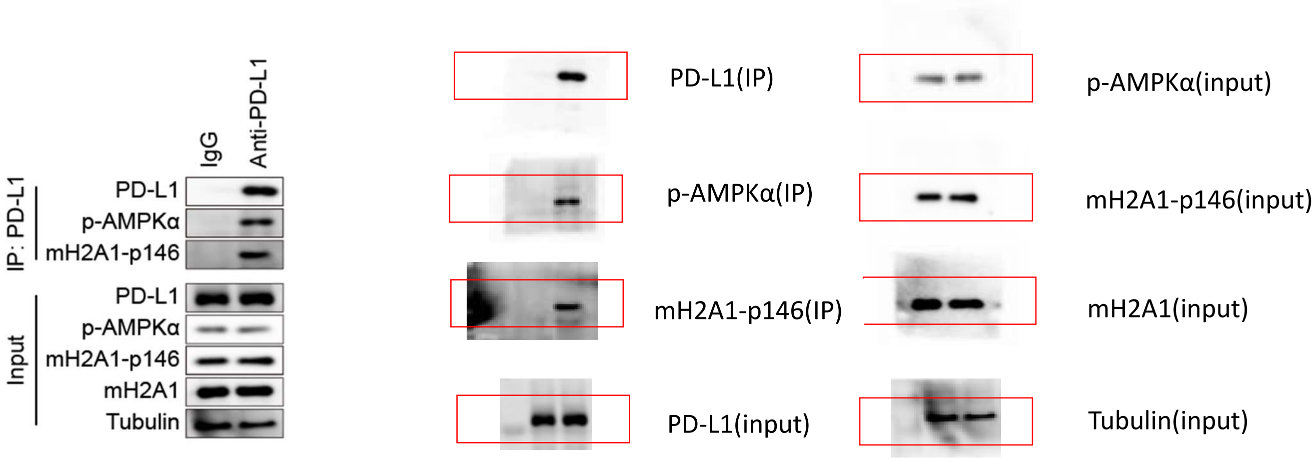

Full unedited gel for Fig. 7G

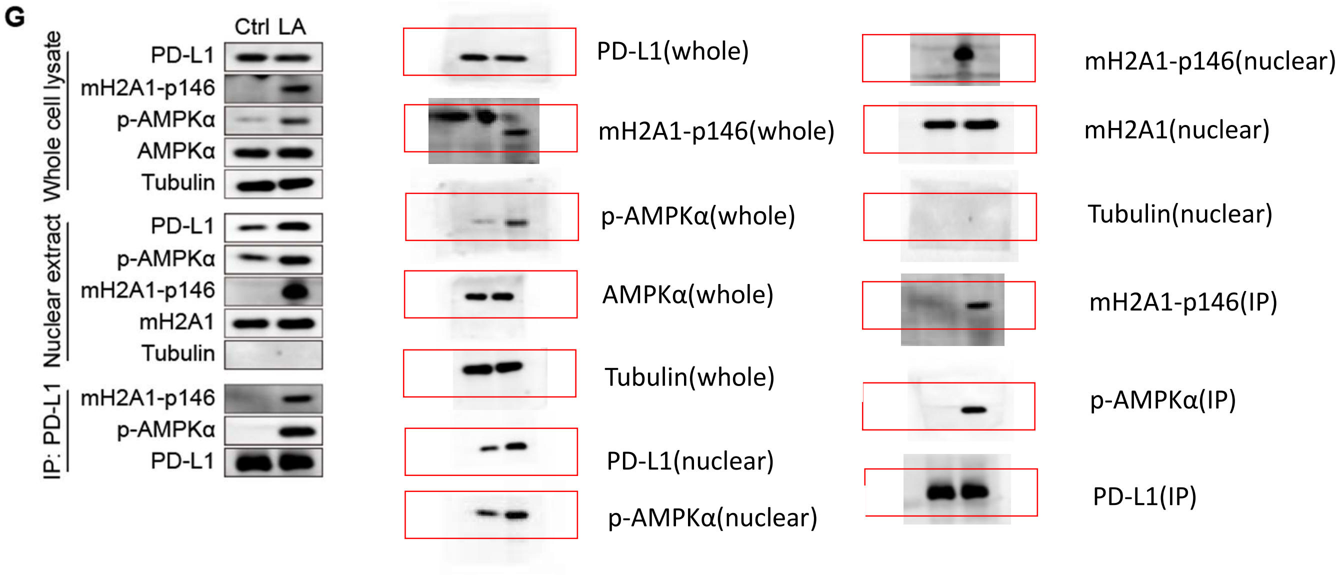

Full unedited gel for Fig. 7H

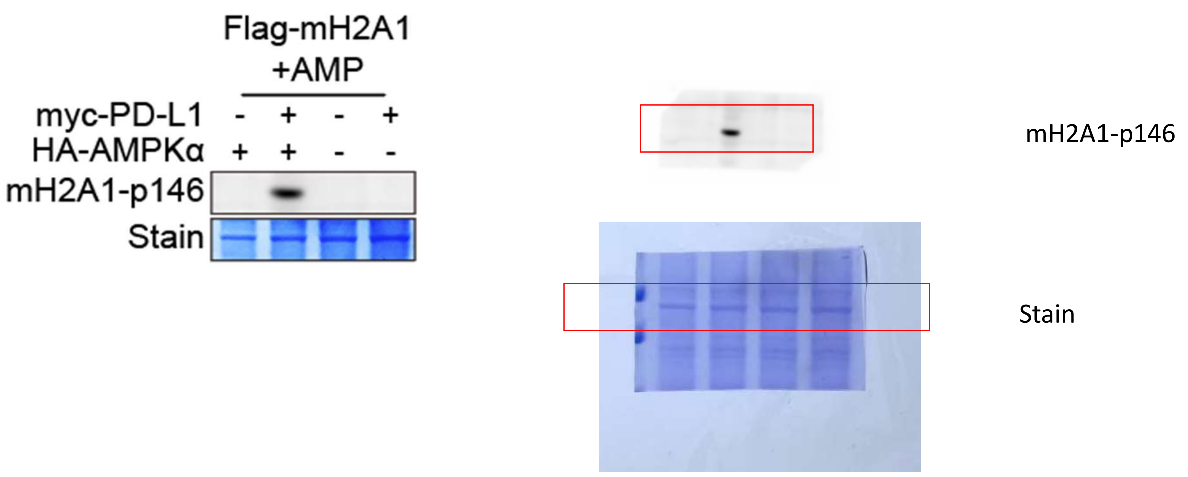

Full unedited gel for Fig. 7I

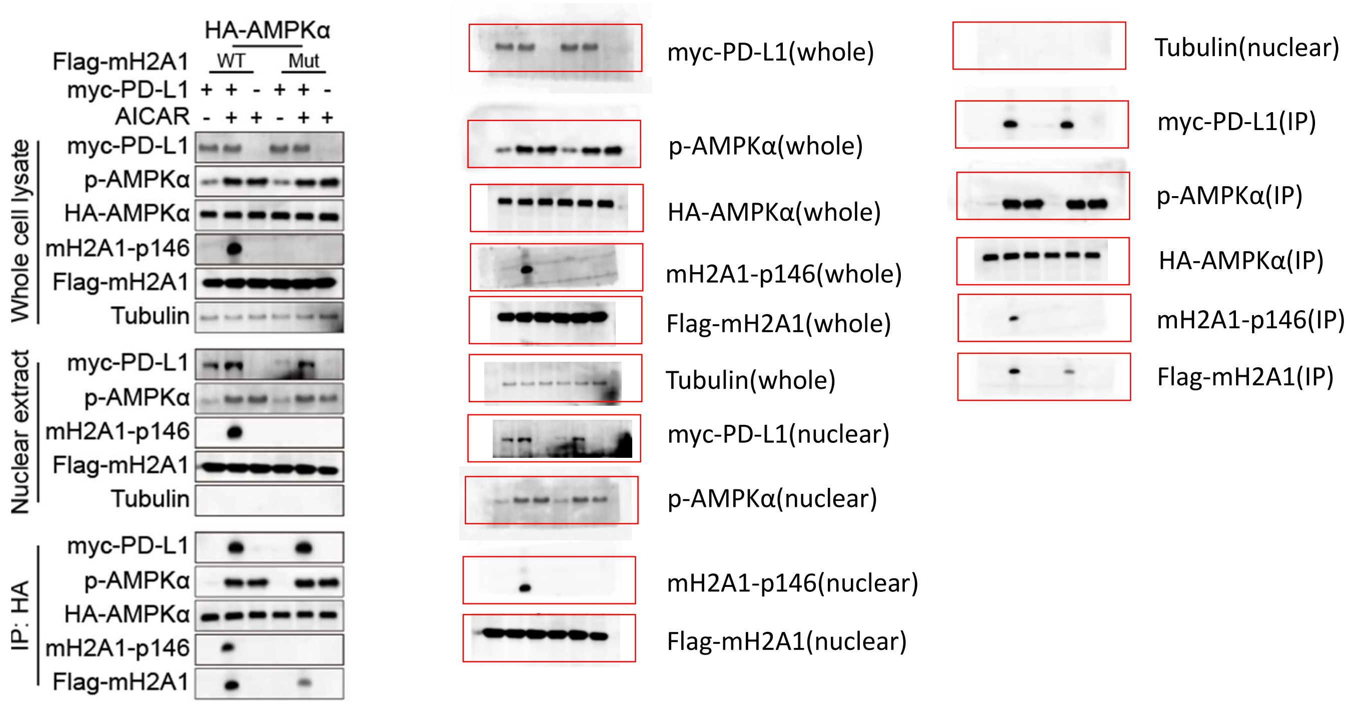

Full unedited gel for Fig. 7J

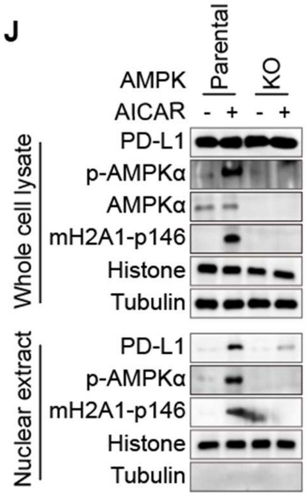

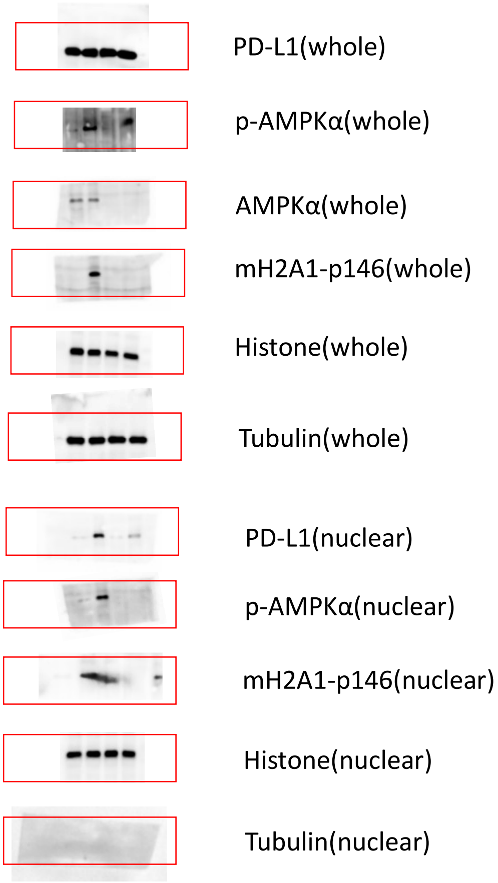

Full unedited gel for Fig. 7K

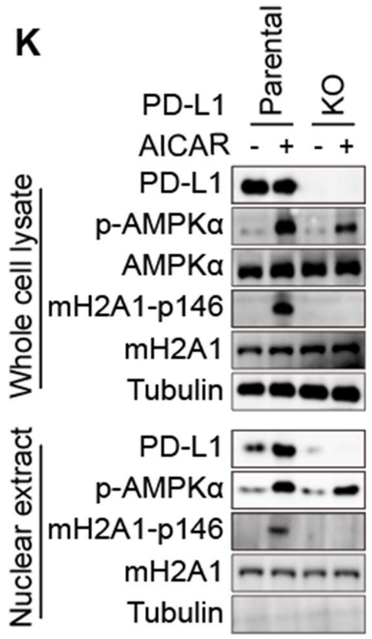

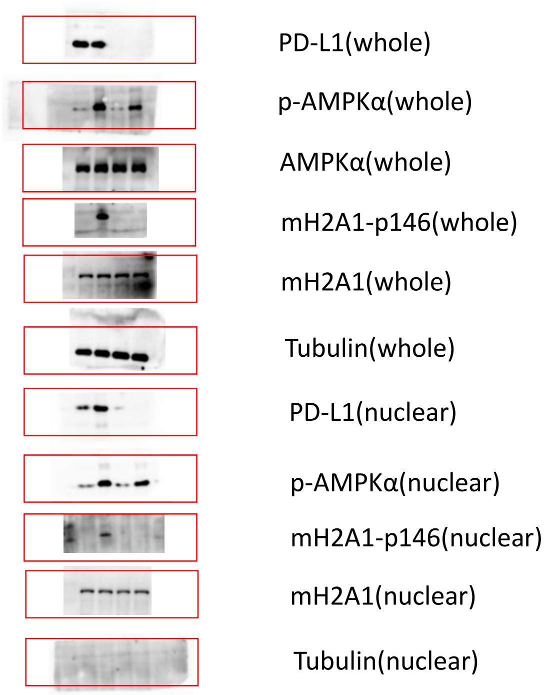

Full unedited gel for Fig. 7L

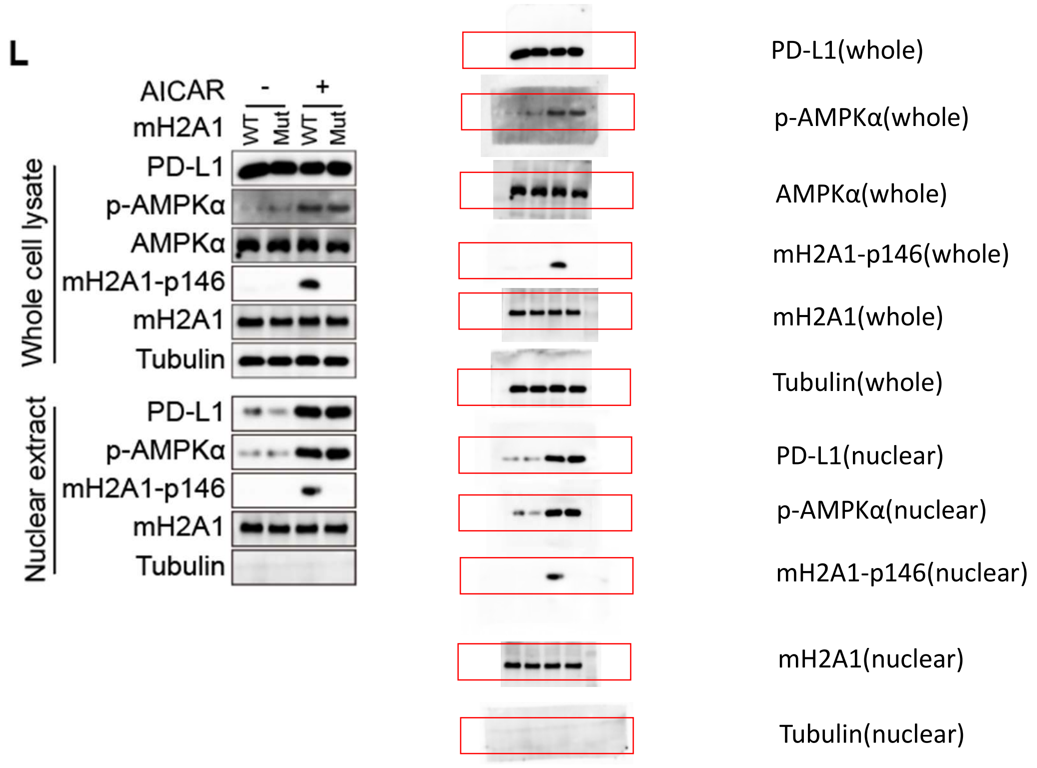

Full unedited gel for Fig. 7N

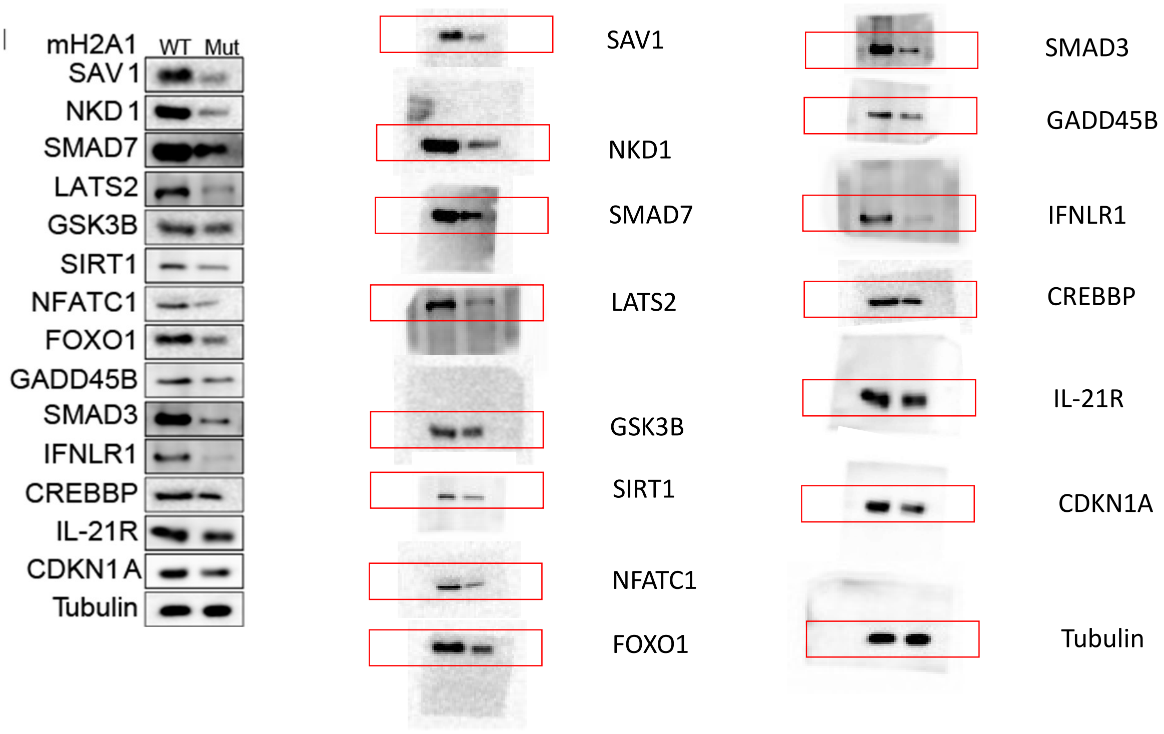

Full unedited gel for Fig. 9A

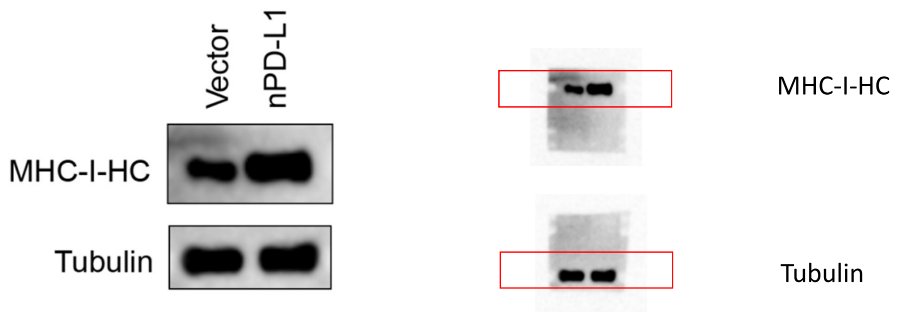

Full unedited gel for Fig. 9B

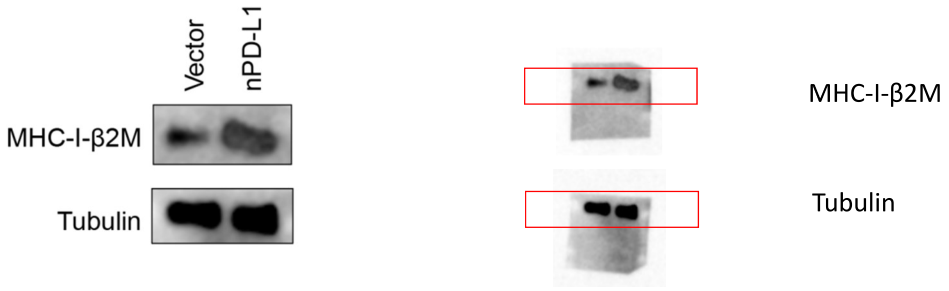

Full unedited gel for Fig. 9D

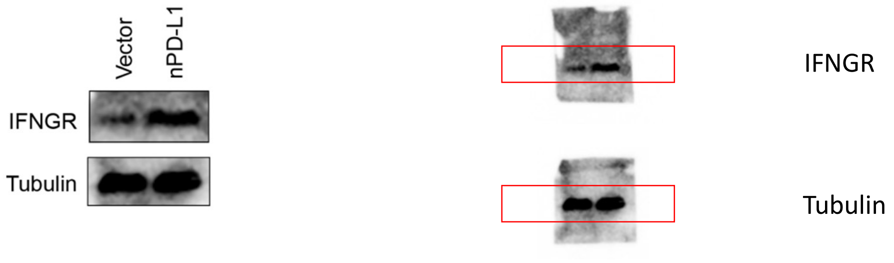

Full unedited gel for Fig. 9F

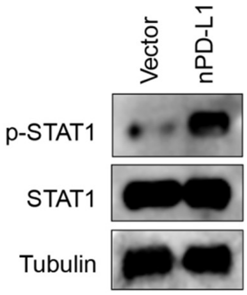

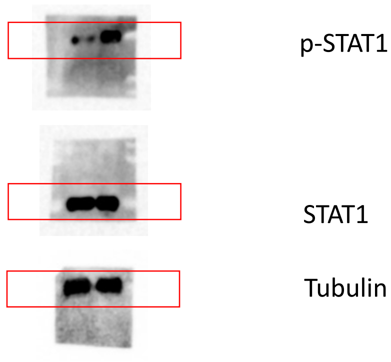

Full unedited gel for Supplemental Fig. 2A

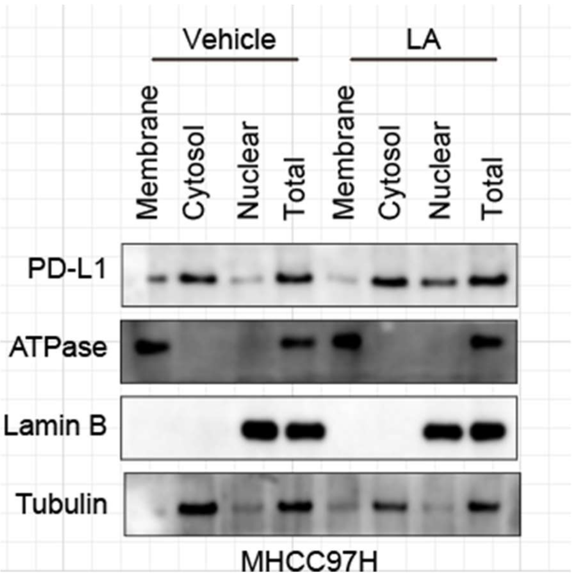

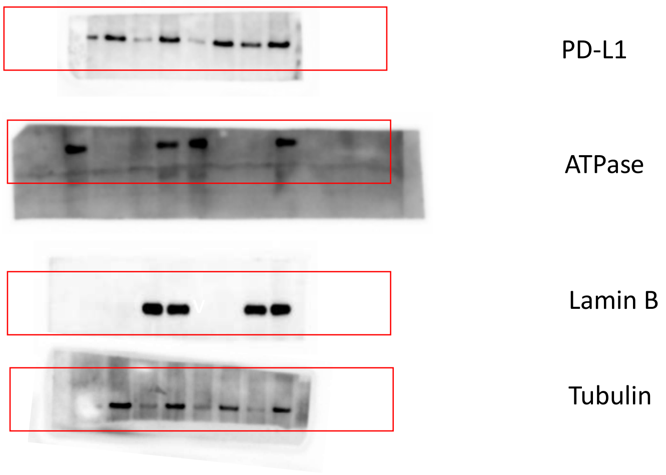

Full unedited gel for Supplemental Fig. 2B

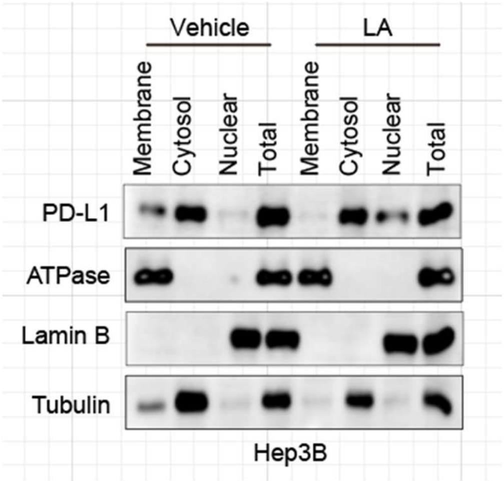

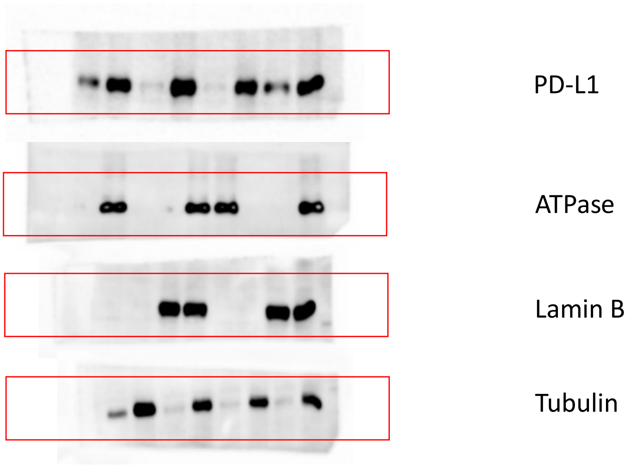

Full unedited gel for Supplemental Fig. 3

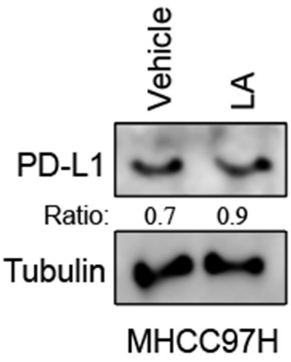

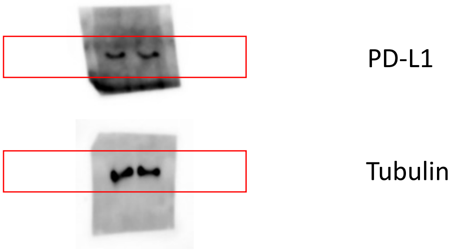

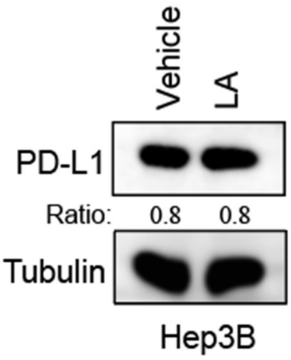

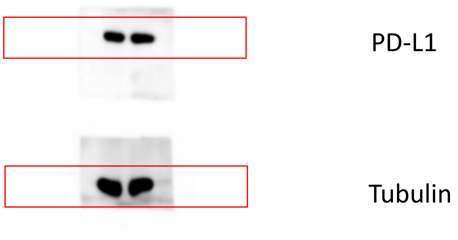

Full unedited gel for Supplemental Fig. 4

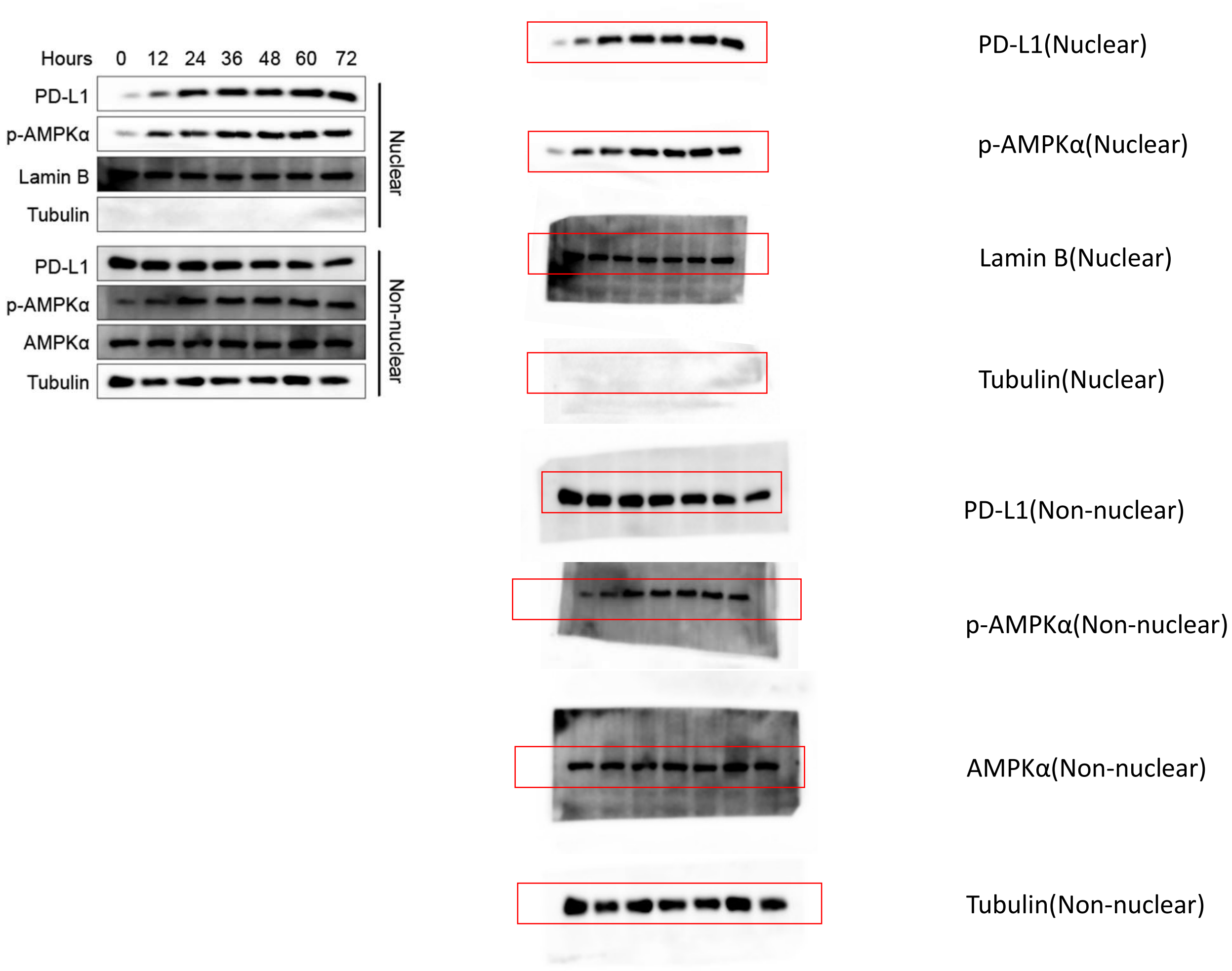

Full unedited gel for Supplemental Fig. 5

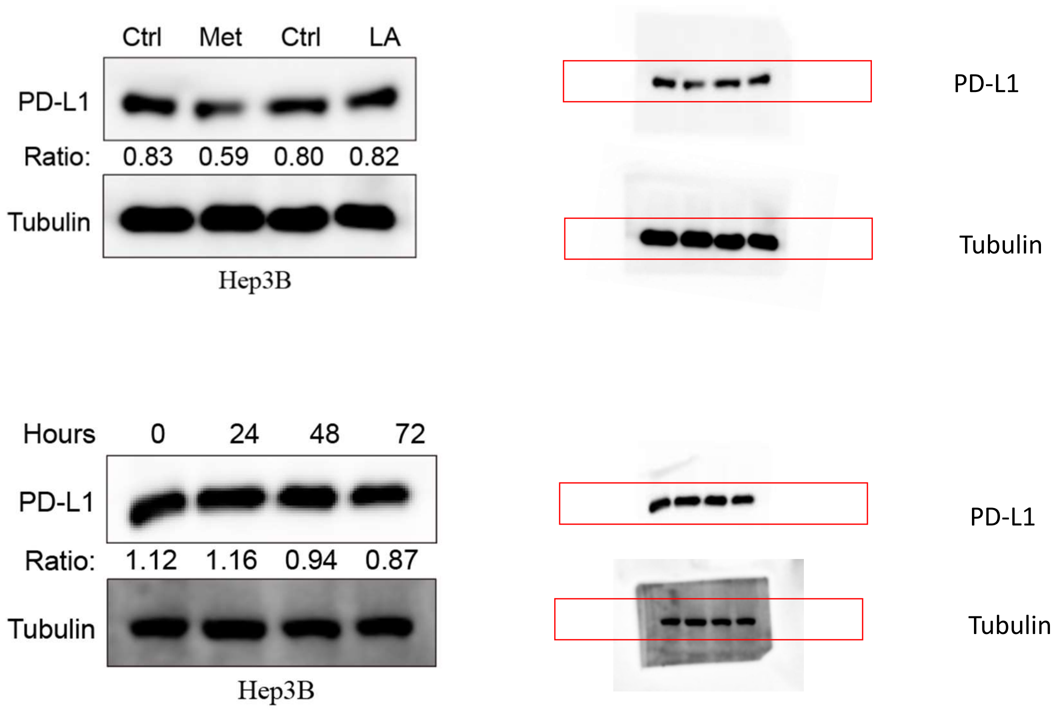

Full unedited gel for Supplemental Fig. 6A

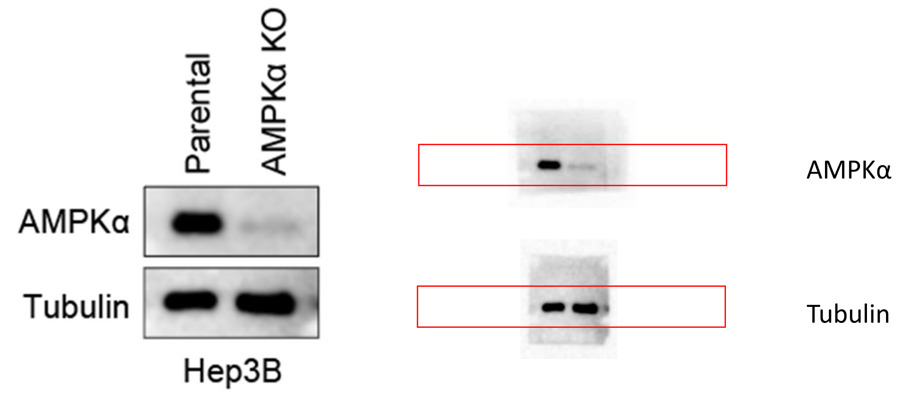

Full unedited gel for Supplemental Fig. 6B

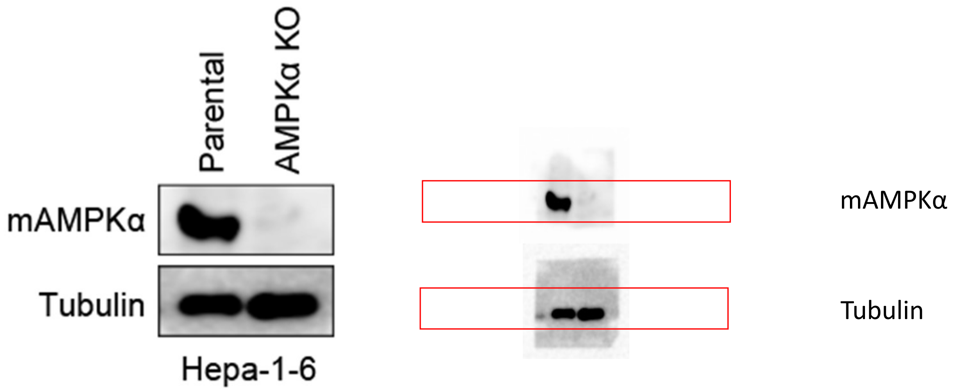

Full unedited gel for Supplemental Fig. 7

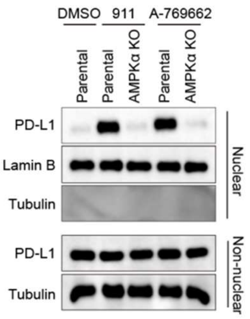

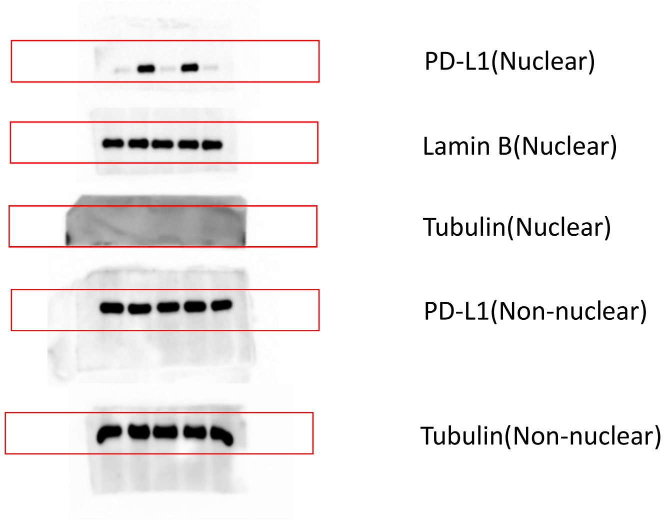

Full unedited gel for Supplemental Fig. 10B

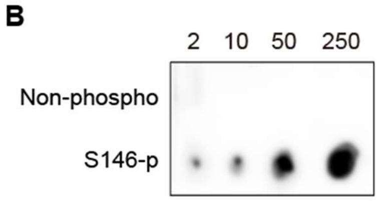

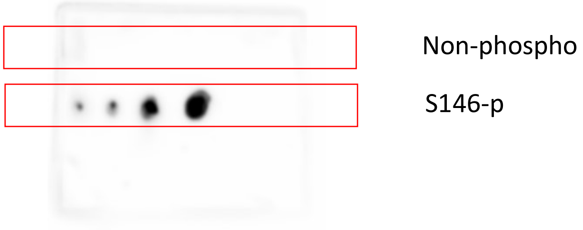

Full unedited gel for Supplemental Fig. 10C

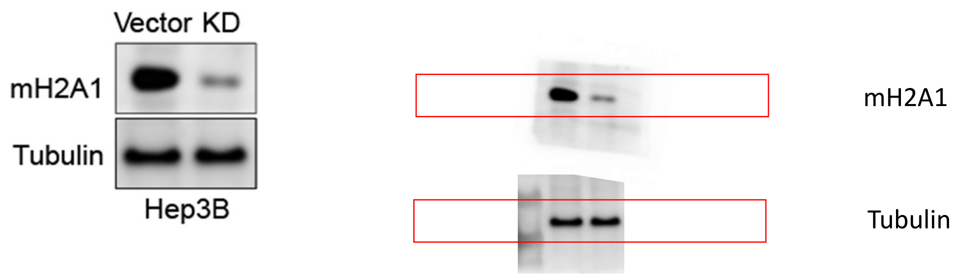

Full unedited gel for Supplemental Fig. 10D

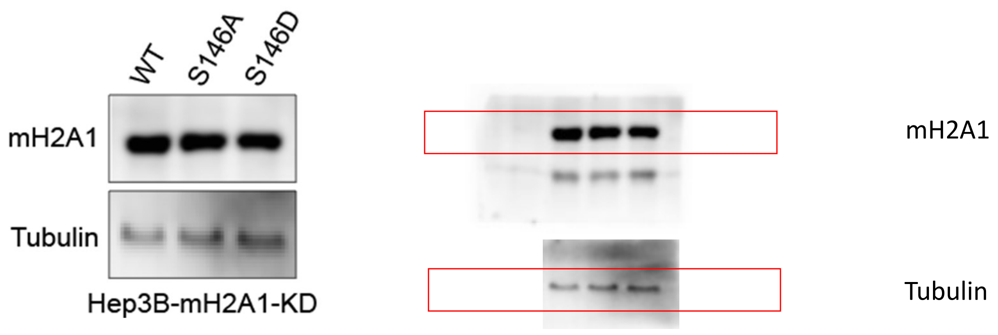

Full unedited gel for Supplemental Fig. 10E

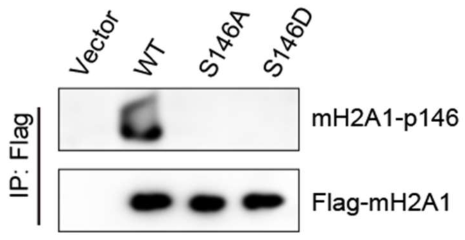

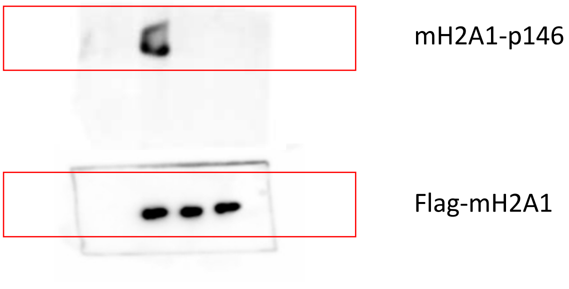

Supplement: Unedited blot and gel images [file jci-134-181314-s146.pdf]
